# Diversity and genomics of bacteriome-associated symbionts in treehopper *Darthula hardwickii* (Hemiptera: Aetalionidae) and implications of their nutritional functions

**DOI:** 10.1128/aem.01738-24

**Published:** 2025-03-04

**Authors:** Xiaohong Han, Jinrui Zhou, Qiong Guo, Christopher H. Dietrich, Lin Lu, Cong Wei

**Affiliations:** 1 Key Laboratory of Plant Protection Resources and Pest Management of the Ministry of Education, Key Laboratory of Integrated Pest Management on Crops in Northwest Loess Plateau of Ministry of Agriculture and Rural Affairs, College of Plant Protection, Northwest A&F University 12469 , Yangling, Shaanxi, China; 2 Illinois Natural History Survey, Prairie Research Institute, University of Illinois Urbana-Champaign 140866 , Champaign, Illinois, USA; Kew Gardens, Surrey, United Kingdom

**Keywords:** sap-feeding insects, *Karelsulcia*, *Ophiocordyceps* fungus, *Tisiphia*, co-evolution, prokaryotic genome evolution

## Abstract

Symbionts play important roles in insect nutritional ecology, and the phylogenies of some vertically transmitted symbionts mirror the host phylogeny. Here we report the diversity, distribution, transmission, and potential functions of symbionts harbored in the aetalionid treehopper *Darthula hardwickii* (Aetalionidae) using multiple methods and compare the potential functions of its obligate symbiont *Karelsulcia* with that of the related aetalionid *Aetalion reticulatum. D. hardwickii* harbors *Karelsulcia* in bacteriomes, a yeast-like fungal symbiont (YLS) in fat bodies, and *Tisiphia* in both the bacteriomes and fat bodies. *Karelsulcia* and YLS are vertically transmitted to the ovaries but do not cluster to form a “symbiont ball” in terminal oocytes, as is the case in other auchenorrhynchan insects. YLS harbored in *D. hardwickii* represents the first known instance of a fungal symbiont being associated with treehoppers. Phylogenetic analysis revealed that Aetalionidae are derived from within Membracidae. Gene truncation and absence were revealed in the tryptophan biosynthetic pathway of *Karelsulcia* from *D. hardwickii*, suggesting this symbiont is no longer capable of providing this essential amino acid (EAA) to its host. Tryptophan is presumed to be supplied to *D. hardwickii* by YLS since tryptophan-related genes are either absent or degraded in *Karelsulcia* and *Tisiphia*. No truncated genes were found in *Karelsulcia* from *A. reticulatum*, but it has lost genes related to the synthesis of other EAAs, as in some leafhoppers. This study sheds new light on the diversity and functions of the nutritional endosymbionts of Membracoidea and processes that may have precipitated symbiont replacement in this diverse insect lineage.

**IMPORTANCE:**

Symbionts in sap-feeding insects play important roles related to nutrition of their hosts, which may change through evolutionary time and vary across host and symbiont lineages. This comparative genomic study indicates that, compared to the related symbionts of other leaf- and treehoppers, the *Karelsulcia* symbiont of the treehopper *Darthula hardwickii* has lost the ability to provide the EAA tryptophan to its host. This function is apparently being performed by a coexisting yeast-like symbiont (YLS). This is the first report of a YLS in a species of treehopper, which suggests that the processes involved in symbiont replacement in treehoppers are similar to those observed in other sap-sucking auchenorrhynchan insects. Phylogenetic analyses of *Karelsulcia* lineages of Membracoidea largely mirror the host insect phylogeny but suggest that Aetalionidae may have originated from Membracidae, in contrast to some recent phylogenies based on the genomic data from the host insects.

## INTRODUCTION

Associations between insects and symbionts are ubiquitous in nature, particularly in insects with nutritionally imbalanced diets such as plant sap, where the symbionts provide essential amino acids (EAAs) and other nutrients (1). Auchenorrhynchan (Hemiptera) insects have specialized organs (i.e., bacteriomes) for harboring the symbiont(s) (2). The common ancestor of Auchenorrhyncha of the order Hemiptera formed a stable symbiosis with the symbiont “*Candidatus* Karelsulcia muelleri” (hereafter referred to as *Karelsulcia*, formerly named *Sulcia*) around 340 million years ago (Ma) (3). The *Karelsulcia* genome has undergone extreme reduction (< 0.3 Mb) but retained genes related to basic cellular functions, genetic information processing, and amino acid synthesis (4, 5). The substantial loss of symbiont genes is attributed to genetic redundancy with their hosts, reduced selection, small population sizes, and frequent population bottlenecks (2, 6).

Dependence on multiple heritable symbionts is very common in auchenorrhynchan insects (7, 8). Besides *Karelsulcia*, auchenorrhynchans usually harbor one or more additional symbionts in their bacteriomes, such as “*Candidatus* Vidania fulgoroideae” (hereafter referred to as *Vidania*) in some planthoppers (9), “*Candidatus* Zinderia insecticola” (hereafter referred to as *Zinderia*) in some spittlebugs (10), “*Candidatus* Hodgkinia cicadicola” (hereafter referred to as *Hodgkinia*) in some cicadas (6), and “*Candidatus* Baumannia cicadellinicola” (hereafter referred to as *Baumannia*) or “*Candidatus* Nasuia deltocephalinicola” (hereafter referred to as *Nasuia*) in some leafhoppers and treehoppers (5, 10). During the diversification of Auchenorrhyncha, the bacteriome-associated symbiont(s) of some species have been lost or replaced by yeast-like fungal symbiont (YLS) residing in the fat bodies, bacteriome sheath, or mycetomes of the host insects (2, 11, 12).

Genomic analyses can deepen our understanding of the nutritional roles of symbionts in their hosts and offer insights into insect–symbiont interactions (4). Such analyses have revealed that *Karelsulcia* and the coresident symbiont(s) can complementarily synthesize all the ten EAAs ( valine, isoleucine, leucine, threonine, lysine, arginine, phenylalanine, tryptophan, histidine, and methionine) for their host insects (9). However, the ability of *Karelsulcia* to produce EAAs varies across major lineages of Auchenorrhyncha, e.g., producing three EAAs (valine, leucine, and isoleucine) in planthoppers (9, 13), seven (valine, isoleucine, leucine, threonine, lysine, arginine, and phenylalanine) in spittlebugs (10), and eight (valine, isoleucine, leucine, threonine, lysine, arginine, phenylalanine, and tryptophan) in cicadas, leafhoppers, and treehoppers (5, 10).

*Karelsulcia* is present in most major lineages of the Membracoidea (3), which includes treehoppers and leafhoppers. However, comparative genomic data for *Karelsulcia* of treehoppers are very limited compared to those available for leafhoppers (5, 14). *Karelsulcia* in a few taxa of Membracoidea, e.g., some species of Ledrinae, has been replaced by YLS (15), but no comparative genomic information is available for such cases, limiting our understanding of the genetic functions of this primary nutritional symbiont and the processes that may lead to symbiont replacement in this group of insects.

Vertical transmission is crucial for maintaining an obligate symbiotic relationship (3, 16). *Karelsulcia* is transmitted strictly through vertical transmission (2, 7). This rigorous mechanism has resulted in the phylogeny of *Karelsulcia* closely mirroring the phylogeny of the host insects (3, 11). Therefore, *Karelsulcia* may help resolve contentious aspects of the phylogeny of its host insects.

The phylogenetic positions of some groups of Membracoidea (e.g., Aetalionidae) are controversial, despite recent phylogenomic analyses (3, 17). A few early phylogenetic studies based on morphological characteristics placed Aetalionidae as the sister to Membracidae (18, 19). Later molecular phylogenetic studies using multiple gene markers (i.e., *18S rDNA*, *28S rDNA*, and *COI*) indicated that Aetalionidae may either be the sister group to Membracidae or have originated from Membracidae (20–22). Recent phylogenomic studies employing anchored hybrid enrichment or transcriptomics have also yielded inconsistent results (17, 23). Notably, a recent study using the phylogeny of *Karelsulcia* to investigate the phylogeny of the host insects based on phylogenomic analyses confirmed that the phylogenetic relationship of *Karelsulcia* is highly congruent with its hosts and suggests that Aetalionidae was derived from within Membracidae (3). However, samples of Aetalionidae in this study were very limited to two species from the Neotropical region (3).

Approximately 38 species of Aetalionidae are distributed in southern North America (e.g., *Aetalion reticulatum*) and the Neotropical region (19). However, one endemic Oriental genus comprising two species (*Darthula hardwickii* and *D. xizangensis*) of Aetalionidae occurs in Yunnan Province and Xizang Autonomous Region of China and neighboring Asian countries (19, 24). Thus, Aetalionidae exhibits a significant geographical gap in distribution, making it an excellent candidate for studying the diversification and phylogeography of auchenorrhynchan insects (in particular Membracoidea) and their co-evolution with related endosymbionts. However, symbionts of this remarkable group remain largely unexplored.

In this study, we investigated the symbionts harbored in the symbiotic organs (bacteriomes and fat bodies) as well as ovaries of *D. hardwickii* using transmission electron microscopy and fluorescence *in situ* hybridization (FISH). The vertical transmission mechanism of symbionts was also investigated. Phylogenetic trees of the symbionts harbored in *D. hardwickii* and its allies were reconstructed to confirm their identities and relationships to the symbionts of other Auchenorrhyncha. We also analyzed the genomes of *Karelsulcia* harbored in *D. hardwickii* and *A. reticulatum,* as well as related symbiont (“*Candidatus* Tisiphia sp.” [hereafter referred to as *Tisiphia*]) co-residing in *D. hardwickii*, to explore their nutritional roles. This is the most detailed investigation, to date, of the nutritional endosymbionts of treehoppers.

## RESULTS

### Composition and distribution of symbionts in the bacteriomes, fat bodies, and ovaries in *D. hardwickii*

The paired, light yellow and translucent bacteriomes were located below the ovaries in the female abdominal cavity (Fig. S1A and B). Each bacteriome was composed of several spherical units (Fig. S1C). The bacteriomes were surrounded by light green fat bodies, and both were connected to the adjacent spiracle by tracheae (Fig. S1B and C).

**Fig 1:**
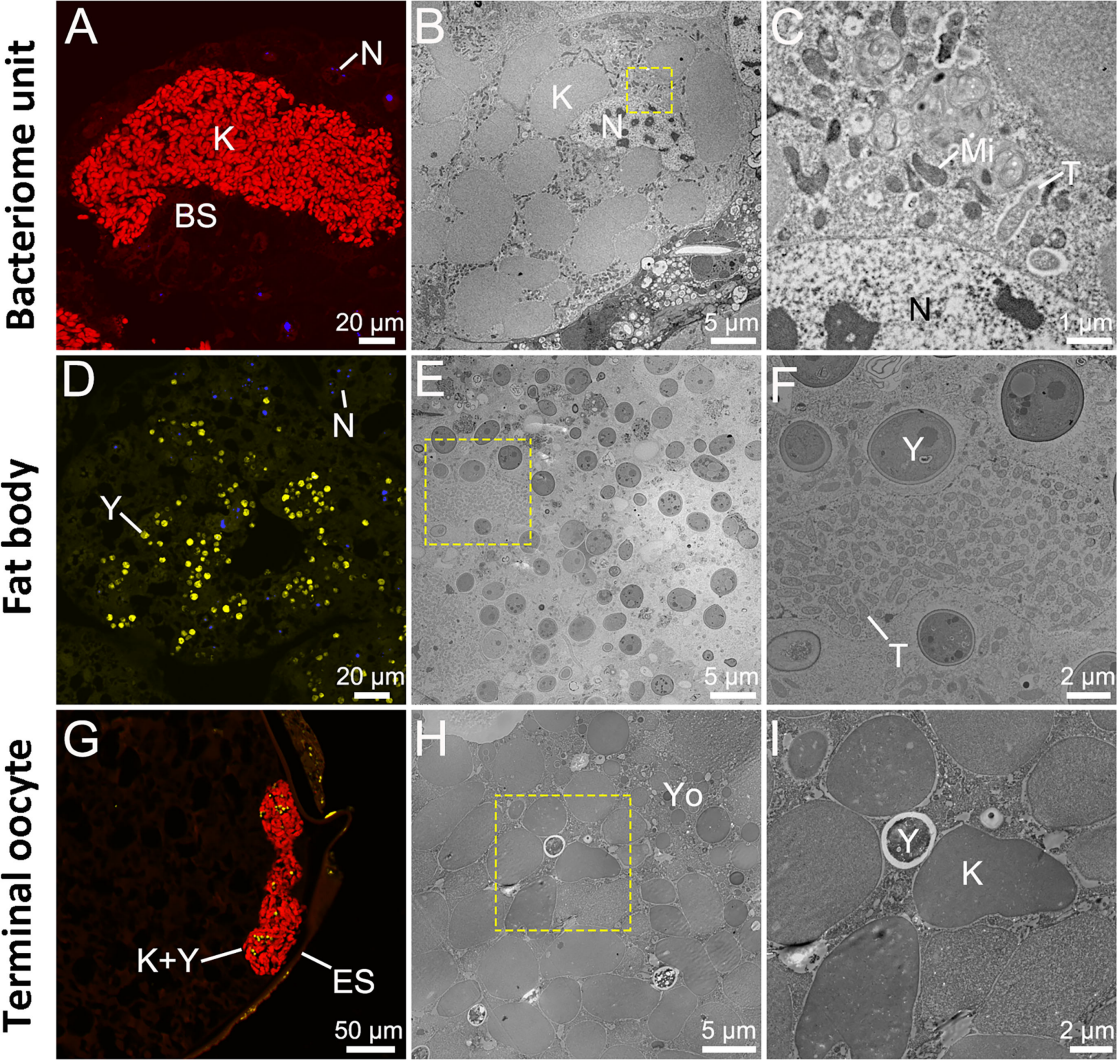
Distribution of symbionts within the bacteriome unit, fat body, and terminal oocyte of *Darthula hardwickii*. (**A, D, G**) Fluorescence microscopic images of the obligate symbionts. Red, yellow, and blue visualize *Karelsulcia*, YLS, and nucleus, respectively. (**B, C**) Ultrastructure of bacteriome unit. (**C**) A magnified image corresponding to the yellow rectangle in (**B**). (**E, F**) Ultrastructure of fat body. (**F**) A magnified image corresponding to the yellow rectangle in (**E**).(**H, I**) Ultrastructure of terminal oocyte. (**I**) A magnified image corresponding to the yellow rectangle in (**H**). Note: Although *Tisiphia* is distributed in both the bacteriomes and fat bodies, it is difficult to visualize by fluorescence *in situ* hybridization. BS, bacteriome sheath; ES, egg shell; K, *Karelsulcia*; Mi, mitochondria; N, nucleus; T, *Tisiphia*; Yo, yolk; Y, YLS.

Diagnostic PCR amplification, fluorescence *in situ* hybridization (FISH) analysis, and ultrastructural observations reveal that *Karelsulcia* is dominant in the bacteriomes and ovaries (Fig. 1A through C and 1G through I), whereas YLS occupies the fat bodies and ovaries (Fig. 1D through F and 1G through I). Diagnostic PCR amplification and ultrastructural observations show that the short rod-shaped *Tisiphia* cells are scattered in both bacteriomes and fat bodies (Fig. 1C and F), but not found in the ovaries (Fig. 1H and I).

**Fig 2:**
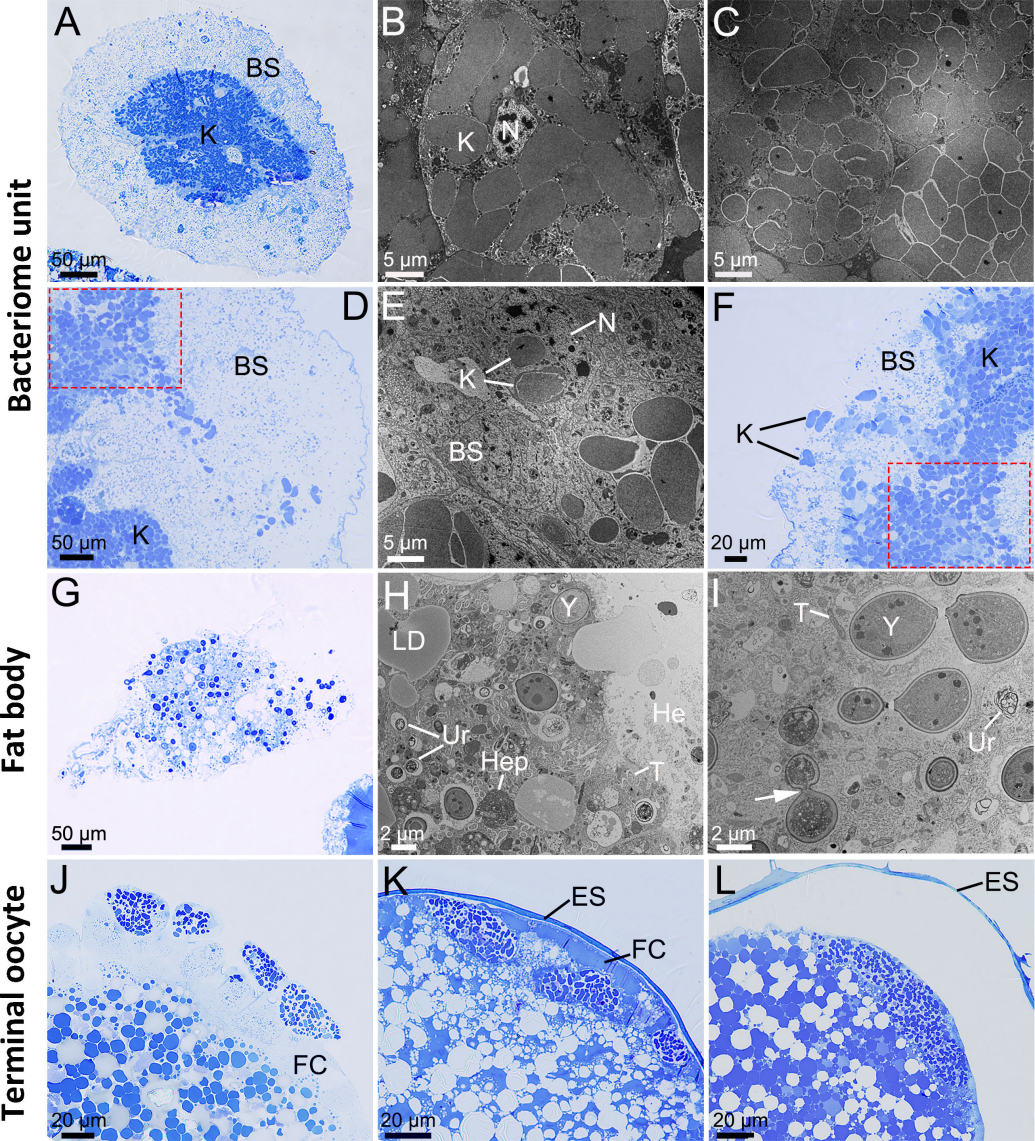
Migration of symbiont cells from the bacteriome unit and fat body to the terminal oocyte in female adults of *Darthula hardwickii*. (**A**) *In vivo* localization of symbionts in the bacteriomes before transmission. (**B**) *Karelsulcia* cells are loosely arranged inside bacteriocytes prior to transmission. (**C**) *Karelsulcia* cells in the transmissive state are tightly arranged outside bacteriocytes. (**D, E**) Enlarged *Karelsulcia* cells are transferred from the intercellular space of bacteriocytes to the bacteriome sheath. (**F**) Enlarged *Karelsulcia* cells are released into the hemolymph. (**G, H**) YLS cells are released from the fat body to the hemolymph. (**I**) YLS is undergoing budding reproduction (white arrow). (**J**) *Karelsulcia* and YLS migrate through the follicle cell into the ovaries. (**K**) *Karelsulcia* and YLS cluster at the perivitelline space. (**L**) *Karelsulcia* and YLS are intermixed within the cluster in the freshly laid eggs. Note: The red dotted rectangles from (**D**) and (**F**) are consistent, and these two images are from adjacent fields of view. BS, bacteriome sheath; ES, egg shell; FC, follicle cell; He, hemolymph; Hep, heterophagosome; K, *Karelsulcia*; LD, lipid droplet; N, nucleus; T, *Tisiphia*; Ur, urocytes; Y, YLS.

### Vertical transmission of symbionts in *D. hardwickii*

In bacteriomes, the *Karelsulcia* cells become swollen, with the membrane becoming more pronounced and irregular, just prior to vertical transmission (Fig. 2A through F). Loosely arranged *Karelsulcia* cells then move from the bacteriocytes to the intercellular space and tightly group there (Fig. 2B and C); some *Karelsulcia* cells then begin to migrate to the cytoplasm of the bacteriome sheath and are finally extruded into the hemolymph (Fig. 2D through F). YLS cells in the fat bodies are released into the hemolymph by splitting of the cell membranes of fat bodies (Fig. 2G through I). When *Karelsulcia* and YLS cells migrate into the terminal oocytes of the ovaries through the cytoplasm of the epithelial plug cells of the ovarioles, they form several clumps at first (Fig. 2J), then gradually accumulate in the perivitelline space (Fig. 2K), and finally cluster at the posterior poles of the terminal oocytes (Fig. 1G and 2L). This state is maintained in the oocytes and is also observed in the eggs after oviposition (Fig. 2L).

### Phylogenomic reconstruction of symbionts

**Fig 3:**
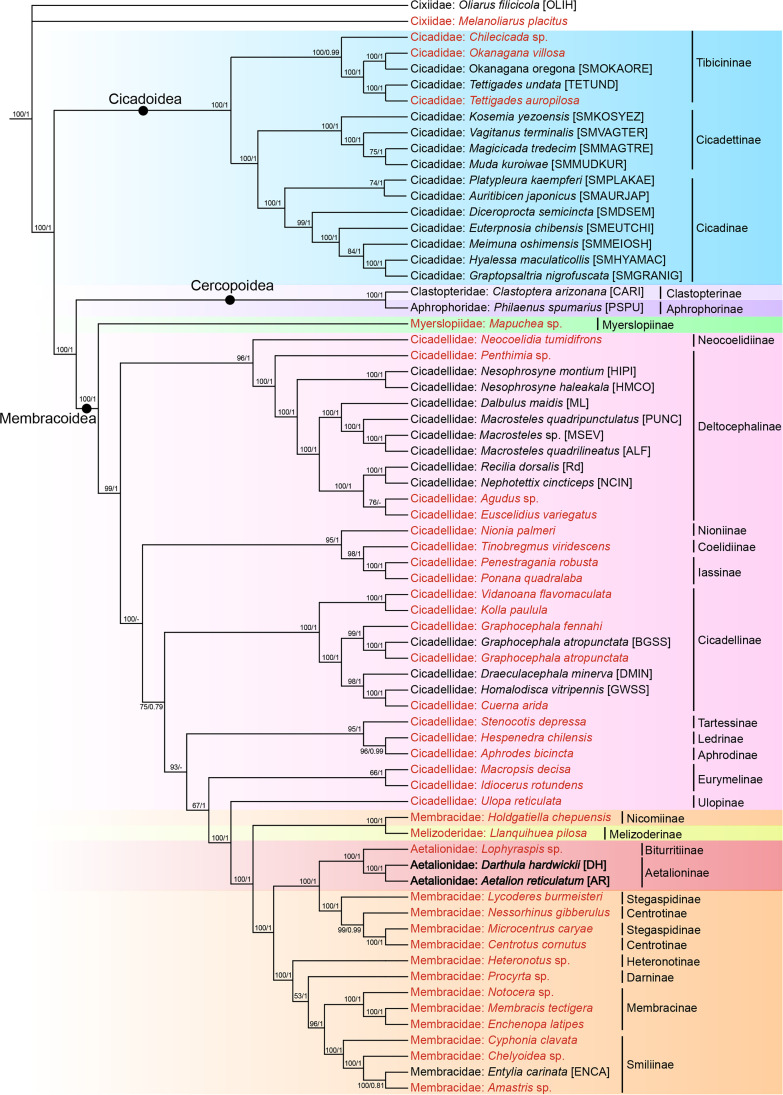
Maximum likelihood phylogeny of *Karelsulcia* based on the 16S rRNA gene and 103 single-copy orthologous genes. Bootstrap support values are indicated on each node in the order of maximum-likelihood/Bayesian inference. Terminals of the branch are labeled with host taxon, host name, and available strain names. *Karelsulcia* lineages of planthoppers are used as outgroups. Labels shown in red font were retrieved from insect transcriptomes. Labels shown in black font were retrieved from complete bacterial genomes. The genome sequences obtained in this study are indicated in bold black.

Both BI and ML phylogenies of *Karelsulcia* constructed based on the concatenated data set of 104 genes (*16S rRNA* +103 single-copy orthologous genes) are basically consistent in topology and have high nodal support overall, with the exception of a few terminal nodes (Fig. 3). The phylogenies indicate that *Karelsulcia* of the three superfamilies (i.e., Cicadoidea, Cercopoidea, and Membracoidea) of Cicadomorpha are all monophyletic with robust support. The *Karelsulcia* lineage of Cicadoidea is sister to the *Karelsulcia* lineage of Cercopoidea and Membracoidea, which are also sister groups (Fig. 3).

Within the major *Karelsulcia* lineage of Membracoidea, the *Karelsulcia* lineage of the sampled species of Myerslopiidae (*Mapuchea* sp.) is sister to the clade comprising all the remaining *Karelsulcia* lineages from Cicadellidae, Melizoderidae, Membracidae, and Aetalionidae. The *Karelsulcia* of Cicadellidae is a paraphyletic group. The monophyly of the *Karelsulcia* lineage of Membracidae is supported, with the exception of the placement of *Holdgatiella* (Membracidae: Nicomiinae) as sister to *Llanquihuea* (Melizoderidae). The *Karelsulcia* lineage of Aetalioninae (represented by *D. hardwickii* and *A. reticulatum*) and that of Biturritiinae (represented by *Lophyraspis* sp.) of Aetalionidae clustered in one clade, which is a sister group to the *Karelsulcia* lineage comprising that of Stegaspidinae and Centrotinae of Membracidae. This suggests that the *Karelsulcia* lineage of Aetalionidae is derived from within the *Karelsulcia* lineage of Membracidae (Fig. 3).

The ML tree of YLSs constructed based on *18S rRNA* shows that YLS of *D. hardwickii* (895 bp) is closely related to the YLS of some leafhoppers and planthoppers (Fig. S2). The BI and ML trees of *Tisiphia* constructed based on the concatenated data set of 365 genes (*16S rRNA* +364 single-copy orthologous genes) show that *Tisiphia* of *D. hardwickii* belongs to the Torix group, which comprises other known *Tisiphia* endosymbionts of insects and is closely related to the *Tisiphia* of *A. reticulatum* (Fig. S3).

### Basic genome features of *Karelsulcia* and *Tisiphia* in *D. hardwickii* and *A. reticulatum*

**Fig 4:**
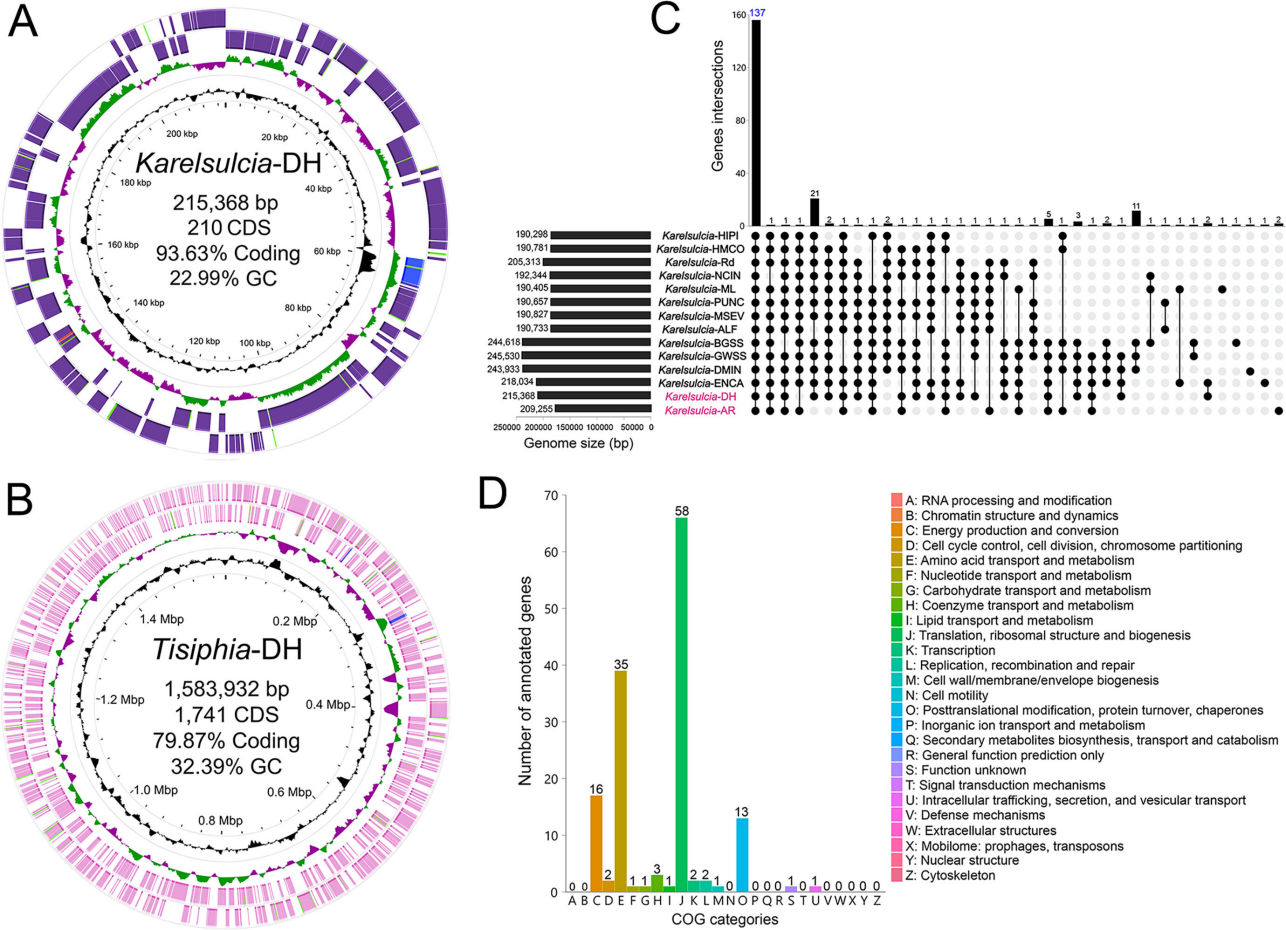
Basic genome features and comparative genomic analysis. (**A, B**) Circular genome maps of *Karelsulcia*-DH and *Tisiphia*-DH. The four circles (outer to inner) represent the following. Circle 1 and 2 exhibit CDS on the forward and reverse strands, as well as rRNA, tRNA, and assembly gap; Circle 3 represents the GC skew ((C − G) / (C + G)) curve (dark green, positive GC skew; violet, negative GC skew); Circle 4 shows the GC content. (**C, D**) Comparative genomic analysis of 14 sampled *Karelsulcia* lineages of treehoppers and leafhoppers. The upset diagram shows the number of core genes, shared orthologous, and unique genes based on the complete genome sequence. The vertical bar plot indicates the number of overlapping genes between different *Karelsulcia* lineages. The black dots, connected by the black lines, represent the collection of these genomes that share the overlapping genes above. The horizontal bar plot indicates the genome size of *Karelsulcia* lineages. (**D**) COG annotation and category assignment based on 137 core genes of 14 sampled *Karelsulcia* genomes.

The genome of *Karelsulcia* in *D. hardwickii* (*Karelsulcia*-DH) comprises a single circular chromosome of 215,368 bp with a 22.99% G + C content (average coverage = 1430.55 × ). It comprises 210 predicted protein-coding genes, one ncRNA, a single rRNA operon, and 26 tRNAs capable of recognizing all amino acid codons (Fig. 4A). The genes of *Karelsulcia*-DH show a wide range of functional categories when annotated to the COG, GO, and KEGG databases (Fig. S4A through C). A COG-based assignment of functional groups reveals that 35.5%, 23.5%, and 11.5% of all protein-coding genes are devoted to translation, ribosomal structure and biogenesis; amino acid transport and metabolism; and energy production and conversion, respectively (Fig. S4A). The genome of *Karelsulcia* in *A. reticulatum* (*Karelsulcia*-AR) is similar, comprising a single circular chromosome of 209,255 bp with a 22.32% G + C content (average coverage = 1300.00 × ) and comprises 197 predicted protein-coding genes, two ncRNAs, a single rRNA operon, and 26 tRNAs capable of recognizing all amino acid codons (Fig. S5A).

The genome of *Tisiphia* in *D. hardwickii* (*Tisiphia*-DH) genome is 1,583,932 bp with a 32.39% G + C content (average coverage = 719.42 × ). This genome comprises 1,741 predicted protein-coding genes, six ncRNAs, a single rRNA operon, and 35 tRNAs (Fig. 4B). The genes of *Tisiphia*-DH show a wide range of functional categories when annotated to the COG, GO, and KEGG databases (Fig. S6A through C). A COG-based assignment of functional groups reveals that 20.1%, 15.0%, and 10.9% of all protein-coding genes are devoted to function unknown; replication, recombination and repair; and translation, ribosomal structure and biogenesis, respectively (Fig. S6A). The genome of *Tisiphia* of *A. reticulatum* (*Tisiphia*-AR) is 1,693,669 bp with a 32.78% G + C content (average coverage = 700.00 × ). This genome comprises 1,509 predicted protein-coding genes, five ncRNAs, a single rRNA operon, and 36 tRNAs (Fig. S5B).

### Comparative genomic analysis of *Karelsulcia* in Membracoidea and the nutritional metabolic potential of *Karelsulcia* in *D. hardwickii*

Using pan-genome analysis, we reannotated and analyzed *Karelsulcia*-DH along with 13 other sampled *Karelsulcia* genomes from representative leafhoppers and treehoppers. Our results show that 137 core genes are shared among all these genomes (Fig. 4C). These 137 core genes are mainly assigned to translation, ribosomal structure and biogenesis; amino acid transport and metabolism; and energy production and conversion (Fig. 4D). Comparative analysis reveals no unique genes in *Karelsulcia*-DH (Fig. 4C), indicating similar functions in *Karelsulcia*-DH compared to the *Karelsulcia* of Membracoidea.

**Fig 5:**
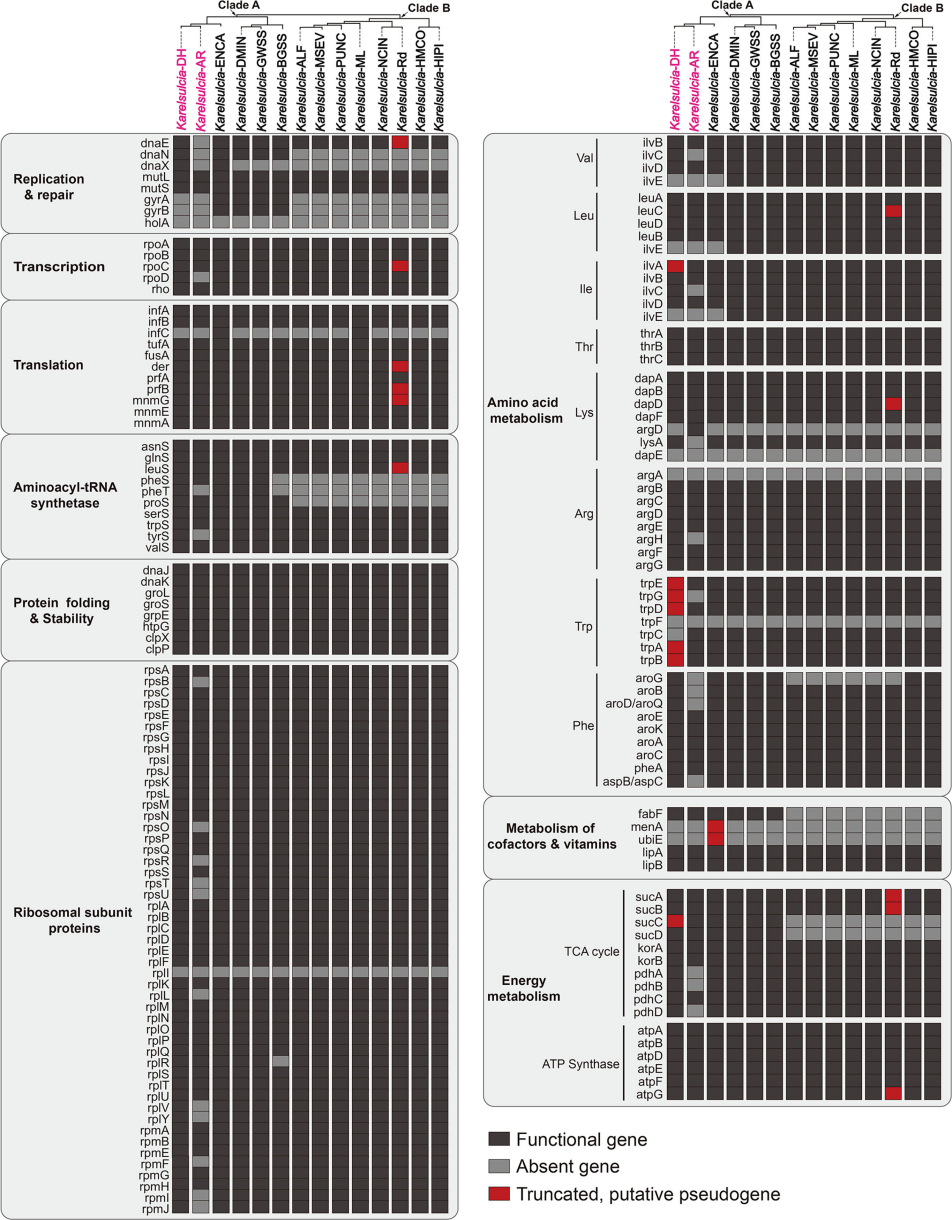
The list of all nonhypothetical protein-coding genes identified in the genomes of 14 sampled *Karelsulcia* lineages of treehoppers and leafhoppers. Black boxes indicate genes that are present, gray boxes indicate genes that are absent, and red boxes indicate genes that are truncated or putative pseudogenized. *Karelsulcia*-DH and *Karelsulcia*-AR obtained in this study are highlighted in pink.

Based on the phylogeny of *Karelsulcia* containing the 14 currently available *Karelsulcia* genomes, we divided *Karelsulcia* of these leafhoppers (11 species) and treehoppers (three species) into two clades (Fig. 5): Clade A, including *Karelsulcia*-DH, *Karelsulcia*-AR, *Karelsulcia*-ENCA, *Karelsulcia*-DMIN, *Karelsulcia*-GWSS, and *Karelsulcia*-BGSS; and Clade B, including *Karelsulcia*-ALF, *Karelsulcia*-MSEV, *Karelsulcia*-PUNC, *Karelsulcia*-ML, *Karelsulcia*-NCIN, *Karelsulcia*-Rd, *Karelsulcia*-HMCO, and *Karelsulcia*-HIPI.

As in other membracoids, the gene content of *Karelsulcia*-DH broadly includes genes involved in genetic information processing (i.e., DNA replication and repair, transcription, and translation) (Fig. 5). *Karelsulcia*-DH retains five genes involved in DNA replication and repair, i.e., DNA polymerase III subunit alpha (*dnaE*), DNA polymerase III subunit beta (*dnaN*), DNA polymerase III subunit gamma (*dnaX*), DNA mismatch repair protein (*mutL*), and DNA mismatch repair protein (*mutS*) and has lost DNA gyrase subunit A (*gyrA*), DNA gyrase subunit B (*gyrB*), and DNA polymerase III subunit delta (*holA*). In contrast, *Karelsulcia*-AR only retains *mutL* and *mutS* and has lost *dnaE*, *dnaN*, *dnaX*, *gyrA*, *gyrB,* and *holA*. The *gyrA* and *gyrB* are retained in the remaining four *Karelsulcia* lineages of Clade A, whereas they are generally absent in Clade B. Additionally, *holA* is also lost in all *Karelsulcia* lineages. The aminoacyl tRNA synthetases related to translation vary within *Karelsulcia* lineages. Similar to other *Karelsulcia* lineages of Clade A (except *Karelsulcia*-BGSS and *Karelsulcia*-AR), *Karelsulcia*-DH retains phenylalanyl-tRNA synthetase alpha chain (*pheS*), phenylalanyl-tRNA synthetase beta chain (*pheT*), and prolyl-tRNA synthetase (*proS*), while these three genes are generally lost in *Karelsulcia* lineages of Clade B. In addition, many truncated genes related to DNA replication and repair, transcription, and translation are present in *Karelsulcia*-Rd (Fig. 5).

The genes related to energy metabolism differ slightly between Clade A and Clade B. The succinyl-CoA synthetase beta subunit (*sucC*) involved in the TCA cycle is truncated in *Karelsulcia*-DH [*sucC*: 279 bp (23%), 186 bp (15%), and 306 bp (26%)] (Fig. 5), which is present in the remaining *Karelsulcia* lineages of Clade A but absent in all the *Karelsulcia* lineages of Clade B. The succinyl-CoA synthetase alpha subunit (*sucD*) is present in *Karelsulcia* lineages of Clade A but absent in that of Clade B. Similar to *Karelsulcia*-DH, 2-oxoglutarate dehydrogenase E1 component (*sucA*) and 2-oxoglutarate dehydrogenase E2 component (suc*B*) of *Karelsulcia*-Rd are also truncated (Fig. 5). Additionally, *Karelsulcia*-AR has lost pyruvate dehydrogenase E1 component subunit alpha (*pdhA*), pyruvate dehydrogenase E1 component subunit beta (*pdhB*), and dihydrolipoyl dehydrogenase (*pdhD*), but these genes are generally present in the remaining *Karelsulcia* lineages.

**Fig 6:**
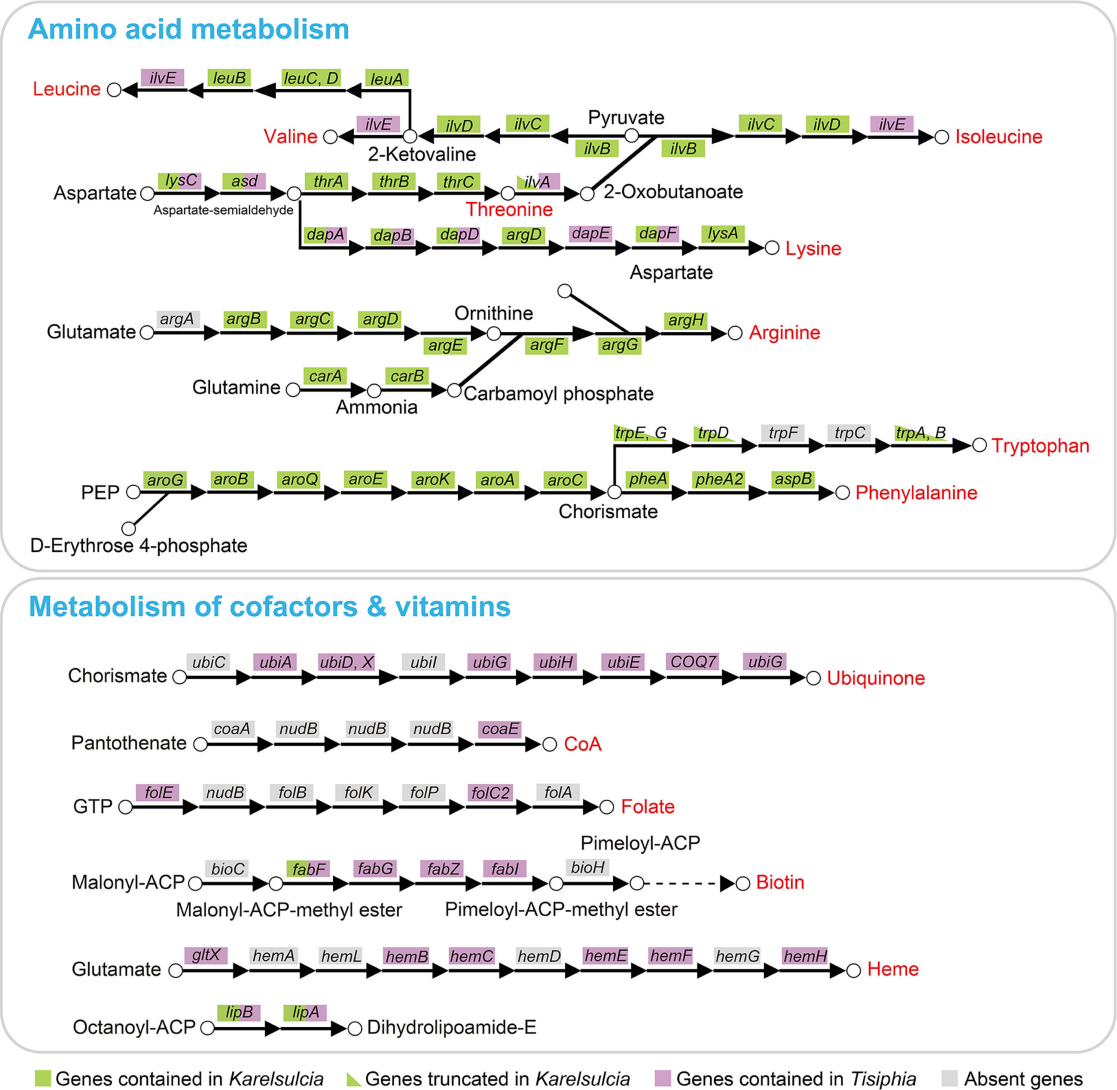
The predicted biosynthesis pathways of EAAs, cofactors, and vitamins in the *Karelsulcia*-DH and *Tisiphia*-DH. Each arrow represents a single step in the reaction, with the abbreviated name of the gene shown above. Green boxes represent genes that are retained in *Karelsulcia*-DH. Half-green boxes represent genes that are truncated in *Karelsulcia*-DH. Pink boxes represent genes that are retained in *Tisiphia*-DH. Gray boxes represent genes that are missing in both. EAAs, cofactors, and vitamins are in red. Chemical compounds involved in some steps are also shown.

The largest difference in the functional content between *Karelsulcia*-DH and all other *Karelsulcia* lineages, including that of the related aetalionid, *Aetalion reticulatum*, is the gene loss and truncation in tryptophan synthesis pathways (Fig. 5 and 6; Fig. S7). *Karelsulcia*-DH has lost two genes, i.e., phosphoribosylanthranilate isomerase (*trpF*) and indole-3-glycerol phosphate synthase (*trpC*), involved in tryptophan biosynthesis; and the remaining five genes, i.e., anthranilate synthase component I (*trpE*), anthranilate synthase component II (*trpG*), anthranilate phosphoribosyltransferase (*trpD*), tryptophan synthase alpha chain (*trpA*), and tryptophan synthase beta chain (*trpB*), are truncated [*trpE*: 375 bp (26%), 321 bp (23%), and 462 bp (33%); *trpD*: 129 bp (23%), 228 bp (40%), and 108 bp (19%); *trpG*: 372 bp (38%) and 507 bp (51%); *trpA*: 249 bp (33%); *trpB*: 105 bp (6%), 273 bp (15%), and 777 bp (43%)] (Fig. 5 and 6; Fig. S7), indicating degradation of these genes. However, *Karelsulcia*-AR only has absent *trpG* and *trpF* and retains intact *trpE*, *trpD*, *trpC*, *trpA*, and *trpB*. Similar to *Karelsulcia*-ENCA and *Karelsulcia*-AR, *Karelsulcia*-DH has lost branched-chain amino acid aminotransferase (*ilvE*) involved in the valine, leucine, and isoleucine biosynthesis, but this gene is retained in *Karelsulcia* of other leafhoppers. Likewise, the threonine dehydratase (*ilvA*) is truncated [*ilvA*: 516 bp (40%), 318 bp (25%), and 276 bp (22%)] (Fig. 5 and 6; Fig. S7). One additional gene cannot definitely be assigned as either acetylornithine deacetylase (*argE*) or succinyl-diaminopimelate desuccinylase (*dapE*), and one gene cannot be assigned with certainty as either arginosuccinate synthase (*argG*) or amino-acid N-acetyltransferase (*argA*) in the lysine and arginine synthesis pathways. We cautiously consider them as *argE* and *argG* in Fig. 5 and 6.

### Nutritional metabolic potential of *Tisiphia* in *D. hardwickii*

*Tisiphia*-DH has a limited number of genes related to the synthesis of EAAs (Fig. 6). It includes branched-chain amino acid aminotransferase (*ilvE*) and threonine dehydratase (*ilvA*) related to valine, leucine, and isoleucine biosyntheses; aspartate kinase (*lysC*) and aspartate-semialdehyde dehydrogenase (*asd*) related to threonine biosynthesis; and 4-hydroxy-tetrahydrodipicolinate synthase (*dapA*), 4-hydroxy-tetrahydrodipicolinate reductase (*dapB*), 2,3,4,5-tetrahydropyridine-2,6-dicarboxylate N-succinyltransferase (*dapB*), succinyl-diaminopimelate desuccinylase (*dapE*), and diaminopimelate epimerase (*dapF*) related to lysine biosynthesis. Some genes, such as *ilvE*, *ilvA,* and *dapE*, coincidentally complement the missing genes of *Karelsulcia*-DH, but many other genes related to EAA synthesis are redundant.

In addition, *Tisiphia*-DH contains a few genes encoding the synthesis of ubiquinone, heme, folate, biotin, and coenzyme A (Fig. 6). *Tisiphia*-DH retains seven genes, i.e., 4-hydroxybenzoate polyprenyltransferase (*ubiA*), 4-hydroxy-3-polyprenylbenzoate decarboxylase (*ubiD*), demethylmenaquinone methyltransferase (*ubiE*), 2-polyprenyl-6-hydroxyphenyl methylase (*ubiG*), 2-octaprenyl-6-methoxyphenol hydroxylase (*ubiH*), flavin prenyltransferase (*ubiX*), and 3-demethoxyubiquinol 3-hydroxylase (*COQ7*), comprising a nearly complete pathway for ubiquinone synthesis. Besides ubiquinone, *Tisiphia*-DH possesses six genes involved in heme synthesis, i.e., glutamyl-tRNA synthetase (*gltX*), porphobilinogen synthase (*hemB*), hydroxymethylbilane synthase (*hemC*), uroporphyrinogen decarboxylase (*hemE*), coproporphyrinogen III oxidase (*hemF*), and coproporphyrin ferrochelatase (*hemH*).

## DISCUSSION

### Conservative distribution and transmission of endosymbionts

Our research indicates that *D. hardwickii* has established symbiotic relationships with *Karelsulcia*, YLS, and *Tisiphia*. As in most other species of Cicadomorpha (2, 15), the ancient symbiont *Karelsulcia* of this treehopper is located in the bacteriomes, while YLS is distributed in the fat bodies. However, in some cicadas that harbor both *Karelsulcia* and YLS, YLS also inhabits the bacteriome sheath besides the fat bodies (2, 11), so further comparative study may reveal a similar variation among treehoppers. The functional implications of such variation remain unclear.

Both *Karelsulcia* and YLS of *D. hardwickii* are vertically transmitted to their offspring. We observed that these symbionts invade and enter the ovaries of females. The transmission process is similar to that of other species of Auchenorrhyncha but has a unique aspect (2, 11). After these symbionts have been transmitted to the ovaries, they enter the yolk but do not cluster to form a “symbiont ball” in the terminal oocytes, and this state is maintained until the eggshell is formed. Whether the formation of symbiotic organs (i.e., bacteriomes) under this transmission state differs from that in other auchenorrhynchans requires further study.

### Nutritional roles of symbionts

As an ancient symbiont, the distribution of *Karelsulcia* is fairly fixed (9). In the present study, we revealed that *Karelsulcia* resides in the bacteriomes of *D. hardwickii*, as is the case in other auchenorrhynchan insects (2, 9, 11). The conserved properties of *Karelsulcia* are evident in its distribution among Auchenorrhyncha lineages as well as in its genome (5, 9). The genome of *Karelsulcia* is highly degraded (4), but *Karelsulcia* lineages associated with different host lineages retain significant homology across Auchenorrhyncha (5, 10). Our comparative study of *Karelsulcia* genomes of *D. hardwickii* and 13 *Karelsulcia* genomes from other Membracoidea revealed that these genomes shared a total of 137 core genes related to basic cellular functions, genetic information processing, and amino acid synthesis, suggesting significant structural and functional conservation across lineages.

Importantly, however, different *Karelsulcia* lineages within Auchenorrhyncha exhibit varying degrees of genome reduction, resulting in differences in the ability to synthesize EAAs (5, 10, 13) and potentially explaining why additional nutritional symbionts have been recruited in some auchenorrhynchan lineages. In the present study, we revealed *Karelsulcia*-DH has lost two genes related to the tryptophan biosynthesis pathway, and the remaining five are truncated, in contrast to the near-complete tryptophan pathways present in the *Karelsulcia* of other investigated auchenorrhynchans (5, 10), including that of the related aetalionid, *A. reticulatum*. This indicates that the tryptophan-related genes in *Karelsulcia*-DH have undergone degradation and that *Karelsulcia*-DH may no longer be able to provide its host with this EAA (25, 26). In this treehopper, tryptophan may be obtained through alternative pathways, e.g., provided by other symbiont(s) (5).

Previous studies revealed that *Karelsulcia* and a coexisting symbiont in the bacteriomes (*Vidania*, *Zinderia*, *Hodgkinia*, *Baumannia,* or *Nasuia*) complementarily produce the ten EAAs for their host insects (5, 6, 9, 10). However, one or more of the bacterial symbionts in some auchenorrhynchan insects have often been replaced by YLS (2, 11, 15, 27). One previous study suggested that YLS in the cicada *Meimuna opalifera* retains the full synthesis pathways of all ten EAAs (2). YLSs are extremely difficult to culture *in vitro* due to co-evolution with their host insect, which makes it challenging to study their genomes. In the present study, we revealed that the betaproteobacterial symbiont (e.g., *Baumannia* or *Nasuia*) was replaced by YLS in *D. hardwickii*, as reported in some leafhoppers (27). However, we were unable to obtain the YLS genome in *D. hardwickii*.

As in obligate symbionts, facultative symbionts may also occupy unique ecological niches in symbiotic relationships (8). However, our understanding of the nutritional roles of facultative symbionts remains quite limited due to their often sparse occurrence among host lineages and host tissues and the concurrent difficulty in obtaining genome sequences. In the present study, we revealed that the facultative symbiont *Tisiphia* co-resides with *Karelsulcia* in the bacteriomes of *D. hardwickii*. Although *Tisiphia*-DH has some genes (e.g., *ilvE*, *ilvA*, and *dapE*) related to synthesis of EAAs, which coincidentally fill the gaps in valine, leucine, isoleucine, and threonine biosynthetic pathways present in *Karelsulcia*-DH, we found no evidence of horizontal transmission of *Tisiphia*-DH; instead, it appears to be a facultative symbiont that spreads via horizontal transmission. Thus, although its genome contains genes related to the synthesis of EAAs, it is not yet known whether *Tisiphia* contributes these nutrients to its host.

As discussed above, YLS potentially has the omnipotent ability to produce all ten EAAs for the host insect (2), with some of its genes being redundant with those of *Karelsulcia*. YLS harbored in *D. hardwickii* represents the first report of a fungal symbiont being associated with treehoppers. Because tryptophan-related genes were not found in *Karelsulcia*-DH or *Tisiphia*-DH, it is reasonable to hypothesize that tryptophan may be supplied stably by YLS to this treehopper species. Previous studies have thoroughly examined the nutritional roles of *Karelsulcia* and its betaproteobacterial partner within Auchenorrhyncha (5, 6, 9, 10), but the complex nutritional roles of YLS have been rarely studied in this group of insects. More comprehensive research will require culturing of YLS and sequencing of their genomes.

### Phylogenomic status of Aetalionidae predicted from *Karelsulcia* phylogeny

Membracoidea is the most diverse superfamily of Auchenorrhyncha, comprising approximately 27,000 described species (28) and including five families: Myerslopiidae, Cicadellidae, Melizoderidae, Membracidae, and Aetalionidae (18, 23). Aetalionidae is a small family within Membracoidea, which includes approximately 40 species of sap-sucking insects (19). This family is divided into two distinct subfamilies: Aetalioninae (with two tribes, Aetalionini and Darthulini) and Biturritiinae (18).

Based on morphological characteristics and some previous molecular phylogenetic analyses, Aetalionidae is regarded as the sister group of Membracidae (18, 19, 23, 28). Nevertheless, the position of Aetalionidae has been unstable among analyses, and some recent results have suggested that Aetalionidae may be derived from within Membracidae (17, 23).

Cao & Dietrich (3) reassembled the transcriptomic data of Skinner et al. (17) to predict and identify orthologs belonging to *Karelsulcia*, and their phylogenetic tree of *Karelsulcia* within Auchenorrhyncha, reconstructed based on *16S rRNA* plus 131 single-copy orthologous genes of *Karelsulcia,* indicated that *Karelsulcia* of Aetalionidae is derived from within Membracidae. This is consistent with our present results.

In the present study, we reconstructed the phylogeny of *Karelsulcia* based on *16S rRNA* and 103 single-copy orthologous genes (loci present for >50% of included species are selected) derived from the *Karelsulcia* genome of *D. hardwickii* and *Aetalion reticulatum* as well as those harbored in other auchenorrhynchan insects whose genomes have been downloaded from NCBI or from Cao & Dietrich (3) since some species included in their phylogenetic data set lack the complete genome of *Karelsulcia*. Our results show that the *Karelsulcia* lineage of Aetalioninae is the sister group of the *Karelsulcia* lineage comprising that of Stegaspidinae and Centrotinae of Membracidae. Although our results indicate that Aetalionidae might be derived from within Membracidae, genomes are available for relatively few representatives of both of these membracoid families, so further analyses including more *Karelsulcia* genomes are needed to resolve the apparent conflicts among results from different phylogenetic studies.

### Conclusion

Our results, combined with those of recent studies, suggest that the *Karelsulcia* endosymbiont of *D. hardwickii* differs from those of other previously studied treehopper and leafhoppers, in that its nutritional role is changing due to the absence and truncation of genes for tryptophan biosynthesis, rendering this endosymbiont incapable of providing this essential amino acid to its host. Our comparative genomic study indicates that the capability of *Karelsulcia* to supply EAAs varies among taxa within Auchenorrhyncha. Tryptophan-related genes are also absent or truncated in both *Karelsulcia* and *Tisiphia* of *D. hardwickii*. Therefore, it is plausible to hypothesize that tryptophan may be consistently supplied by YLS to this treehopper. Interestingly, the near-complete tryptophan pathways are present in the *Karelsulcia* of *A. reticulatum*. The *Karelsulcia* genome of *D. hardwickii* (*Darthula*) is relatively degraded compared to that of *A. reticulatum* (*Aetalion*), which might partly explain why there are so few aetalionids in the Oriental region and sheds new light on the co-diversification of this primarily nutritional endosymbiont with its host insects. Future research incorporating *Karelsulcia* genomes from additional treehoppers is needed to more completely reveal the patterns and extent of symbiont genome degradation in this lineage as they relate to replacement of ancient bacterial endosymbionts by YLS and other symbionts.

## MATERIALS AND METHODS

### Sample collection and tissue dissection

During the adult emergence period, adults of *D. hardwickii* were captured in Pianma Town (26°0’N, 98°37’E), Lushui City, Yunnan Province, China and Dawei Mountain National Nature Reserve (22°54’N, 103°41’E) of Yunnan Province, China, in June of both 2023 and 2024. Before dissection, *D. hardwickii* was briefly anesthetized at 4°C for 5 minutes, externally sterilized with 75% ethanol, and then rinsed with sterile water five times. Dissection was performed under a stereoscopic zoom microscope (Motic SMZ168; Xiamen, China) in sterile conditions with sterile forceps to obtain intact bacteriomes, fat bodies, and ovaries. The dissected samples were preserved in centrifuge tubes containing paraformaldehyde (4%) for fluorescence microscopy, those containing glutaraldehyde (2.5%) for transmission electron microscopy, and 100% alcohol for DNA extraction, respectively, for further study. Internal morphology was photographed with a digital camera (Canon 550D, Japan). In addition, one female adult of *A. reticulatum* collected from Misiones (25°37’S, 54°32’W), Argentina, in January of 2008 and preserved in 100% ethanol was used for DNA extraction.

### Fluorescence *in situ* hybridization

The bacteriomes, fat bodies, and ovaries of female adults (20 individuals) were fixed in paraformaldehyde (4%), dehydrated in a graded ethanol series, cleared four times in xylene for 2 hours, and finally embedded in melted paraffin. Paraffin blocks were sectioned to 4 µm. Thin sections were used for fluorescence microscopy. The FISH assay was performed as previously described (29, 30). The probe sequences were Cy3-CCACACATTCCAGTTACTCC for *Karelsulcia* (6) and Cy5-CCTGCCTGGAGCACTCT for YLS (2) (Table S1). Briefly, analysis of each slide was carried out using a final volume of 25 µL of hybridization buffer, which contained 0.25% bovine serum albumin (BSA), 2.5 × SSC (1 × SSC is 0.15 M NaCl plus 0.015 M sodium citrate), 12.5% dextran sulfate, and fluorescently labeled probes at 200 nM. Hybridization was performed overnight at 37°C in a humidified chamber. Slides were eventually observed and imaged under a confocal microscope (Olympus FV 3000, Japan) (11, 30).

### Transmission electron microscopy

The bacteriomes, fat bodies and ovaries (20 individuals) stored in glutaraldehyde (2.5%) were fixed overnight at 4°C. After rinsing with phosphate-buffered saline (PBS) five times, the samples were then postfixed with 1% osmium tetroxide (OsO_4_) in 0.1 M PBS for 1.5 hours. After washing with PBS six times, the samples were dehydrated in a graded ethanol series (30%, 50%, 70%, 80%, and 90% for 10 minutes each; 100% for 30 minutes, repeated four times). Next, the samples were infiltrated with a graded mixture of ethanol and White London resin (LR-White) (Sigma-Aldrich, USA), subsequently infiltrated with LR-White for 24 hours twice, and finally embedded in pure LR-White and polymerized at 55°C for 48 hours (11).

Semi-thin sections (700 nm) were cut using an ultramicrotome (EMUC7, Leica, Austria) with a glass knife, stained with 1% methylene blue, and photographed under a DM6 B light microscope (Leica, Germany). Ultrathin sections (70 nm) were cut using a diamond knife on the ultramicrotome, doubly stained with uranyl acetate and lead citrate, and finally examined under transmission electron microscopy (Tecnai G2 Spirit Bio, FEI, Czech Republic).

### DNA extraction and diagnostic PCR analyses

Genomic DNA of bacterial symbionts of *D. hardwickii* (15 individuals) and *A. reticulatum* (one individual) were extracted using QIAmp DNA Mini Kit (QIAGEN, Germany), and fungal symbionts of *D. hardwickii* (15 individuals) were extracted using the E. Z. N. A. HP Fungal DNA Kit (Omega Bio-Tek, USA). Diagnostic PCR amplification was performed to confirm the presence of dominant symbionts in the bacteriomes, fat bodies, and ovaries. PCR primers and annealing temperatures used for the amplification of *Karelsulcia*, *Tisiphia*, and YLS are listed in Table S2. PCR amplification was carried out under the following cycling conditions: an initial denaturation step at 94°C for 5 minutes, followed by 30 cycles of 94°C for 30 seconds, annealing at different temperatures of different sequences for 1 minute, and 72°C for 1 minute, followed by a final extension step at 72°C for 5 or 10 minutes. The PCR products were determined by 1% agarose gel electrophoresis and then purified with a universal DNA purification kit (Hlingene, China). PCR products were sent for sequencing to Sangon Biotech Co., Ltd. (Shanghai, China).

### Genome sequencing, assembly, and annotation

The bacterial symbionts obtained in this study were subjected to next-generation sequencing using the TruSeqTM DNA Sample Prep Kit (Illumina, USA) and the DNBSEQ platform (paired-end, 2 × 150 bp). Using Fastp v0.23.1 (31) with default settings, we performed quality control on the data, filtering out low-quality reads. The high-quality data were *de novo-*assembled using A5-MiSeq (32) and SPAdes v3.7.1 (33) to generate contigs and scaffolds. The resulting assemblies were evaluated and compared, and base correction was performed using Pilon v1.24 (34). We obtained species information for the genomes by aligning the genome sequences with the Nucleotide Sequence Database (NT).

To improve the accuracy of gene translation site identification and reduce the false positive rate in gene prediction, protein-coding genes of genomes were predicted using GeneMarkS v4.32 (35). The tRNA genes across the whole genome were predicted using tRNAscan-SE v2.0 (36). The rRNA genes were predicted using Barrnap v0.9 (https://github.com/tseemann/barrnap). The prediction of other noncoding RNAs was mainly achieved by comparison with the Rfam v14.1 database (37).

GO annotation for protein-coding genes was performed using BLAST2GO software v1.0 (38) with default parameters. For COG annotation, we used Diamond v2.0.14 (http://github.com/bbuchfink/diamond) and Blastp v2.5.0 (https://blast.ncbi.nlm.nih.gov/Blast.cgi) to align the protein-coding gene sequences with protein sequences in the eggNOG (COG) database. We selected a sequence alignment threshold of 1e^−6^ and assigned the eggNOG identifier of the best hit to the corresponding protein-coding gene. KO and pathway annotations were mainly carried out using the KEGG’s KAAS automated annotation system (v2.1) (39). The gene set was selected as “For Prokaryotes,” and the annotation method was set to “blast” using the bi-directional best hit (BBH) rule for gene KO determination. After annotation, we verified the accuracy of the annotations using Prokka v1.14.6 (40). Additionally, the gene circle was generated using Proksee (https://proksee.ca/) (41).

### Phylogenetic analysis

In addition to genomes (genes) obtained in this study, genomic data of *Karelsulcia* of 67 representative species were downloaded from the National Center for Biotechnology Information (NCBI) or from Cao & Dietrich (3) (see Table S3 for details). Genomic data of *Tisiphia* and its allies of 28 representative species were downloaded from NCBI (see Table S4 for details). Sequences of *18S rRNA* of *Ophiocordyceps* and its allies of 29 representative species were also downloaded from NCBI (see Table S5 for details).

Universal single-copy orthologs (USCOs) were extracted from genomes with BUSCO v.5.4.7 (42) against the reference gene set of Flavobacteriales (number of BUSCOs = 733) and Rickettsiales (number of BUSCOs = 364). We modified the default standard deviations (σ) of the mean USCO length to 2σ to classify more USCOs as “complete” because the BUSCO pipeline did not output incomplete “fragmented” USCO sequences. USCO nucleotide sequences were used for subsequent analyses. Nucleotide sequences of each locus were aligned using MAFFT v7.450 (43) with the L-INS-I method. To reduce compositional heterogeneity, alignments were trimmed using TrimAl v1.4.1 (44) with the parameters “automated1.” We generated the USCO matrices and chose the 50% similarity matrix to reconstruct the phylogenetic tree of *Karelsulcia* and *Tisiphia*. We used IQ-Tree v2.2.2.7 (45) with the built-in ModelFinder (46) to automatically evaluate substitution models. For the phylogeny of *Karelsulcia* and *Tisiphia*, the best model selected according to the Bayesian Information Criterion (BIC) was GTR + F + I, which was used to construct the ML tree. This model was also used with MrBayes v3.2.7a (47) for the BI tree. For the phylogeny of fungal symbionts, the best model selected according to BIC was K2*P* + G4, which was used to construct the ML tree. Finally, the tree files were visualized and refined using iTOL v6 (48).

### Comparative genomic analyses

Pan-genome analysis was conducted to compare 14 sampled *Karelsulcia* genomes (i.e., *Karelsulcia*-DH, *Karelsulcia*-AR, *Karelsulcia*-ENCA, *Karelsulcia*-DMIN, *Karelsulcia*-GWSS, *Karelsulcia*-BGSS, *Karelsulcia*-ALF, *Karelsulcia*-MSEV, *Karelsulcia*-PUNC, *Karelsulcia*-ML, *Karelsulcia*-NCIN, *Karelsulcia*-Rd, *Karelsulcia*-HMCO, and *Karelsulcia*-HIPI) from Membracoidea, aiming to identify the core genes, shared orthologous, and unique genes of the *Karelsulcia* genomes. We used the annotation results obtained from Prokka v1.14.6 (40) for pan-genome analysis with Roary v3.11.2 (49). For visualization of truncated genes, we used MEME v5.5.5 (50) to search for motifs in truncated genes to detect conserved and variable motifs.

## Data Availability

Raw Illumina reads generated for this study were submitted to the Sequence Read Archive (SRA) database of NCBI under BioProject number PRJNA1150132 (bacteriome metagenome of *Darthula hardwickii*) and PRJNA1185029 (abdomen metagenome of *Aetalion reticulatum*). The Whole-Genome Shotgun project has been deposited at GenBank under accession numbers CP168933 (*Karelsulcia*-DH), JBHFQD000000000 (*Tisiphia*-DH), JBJHFL000000000 (*Karelsulcia*-AR), and JBJHFM000000000 (*Tisiphia*-AR). Fragment genes of symbiont (*16S rDNA* and *18S rDNA* of *D. hardwickii*) are available under accession numbers PQ147044 (*Karelsulcia*), PQ149853 (*Tisiphia*), and PQ147053 (YLS). Sequences of other symbionts and its allies were downloaded from NCBI or from Cao & Dietrich (3) (see Tables S3 to S5 for details).

## SUPPLEMENTAL MATERIAL

Supplemental material aem.01738-24-s0001.docx Figures S1 to S7; Tables S1 to S5.

ASM does not own the copyrights to Supplemental Material that may be linked to, or accessed through, an article. The authors have granted ASM a non-exclusive, world-wide license to publish the Supplemental Material files. Please contact the corresponding author directly for reuse.
