# Supplementary material for: Diversity and genomics of bacteriome-associated symbionts in treehopper Darthula hardwickii (Hemiptera: Aetalionidae) and implications of their nutritional functions
Source: Appl Environ Microbiol. 2025 Mar 4;91(4):e01738-24. doi: 10.1128/aem.01738-24 (PMC12016543; doi:10.1128/aem.01738-24)
Supplement: Supplemental material — Figures S1 to S7; Tables S1 to S5. [file aem.01738-24-s0001.docx]

**Supplemental materials**


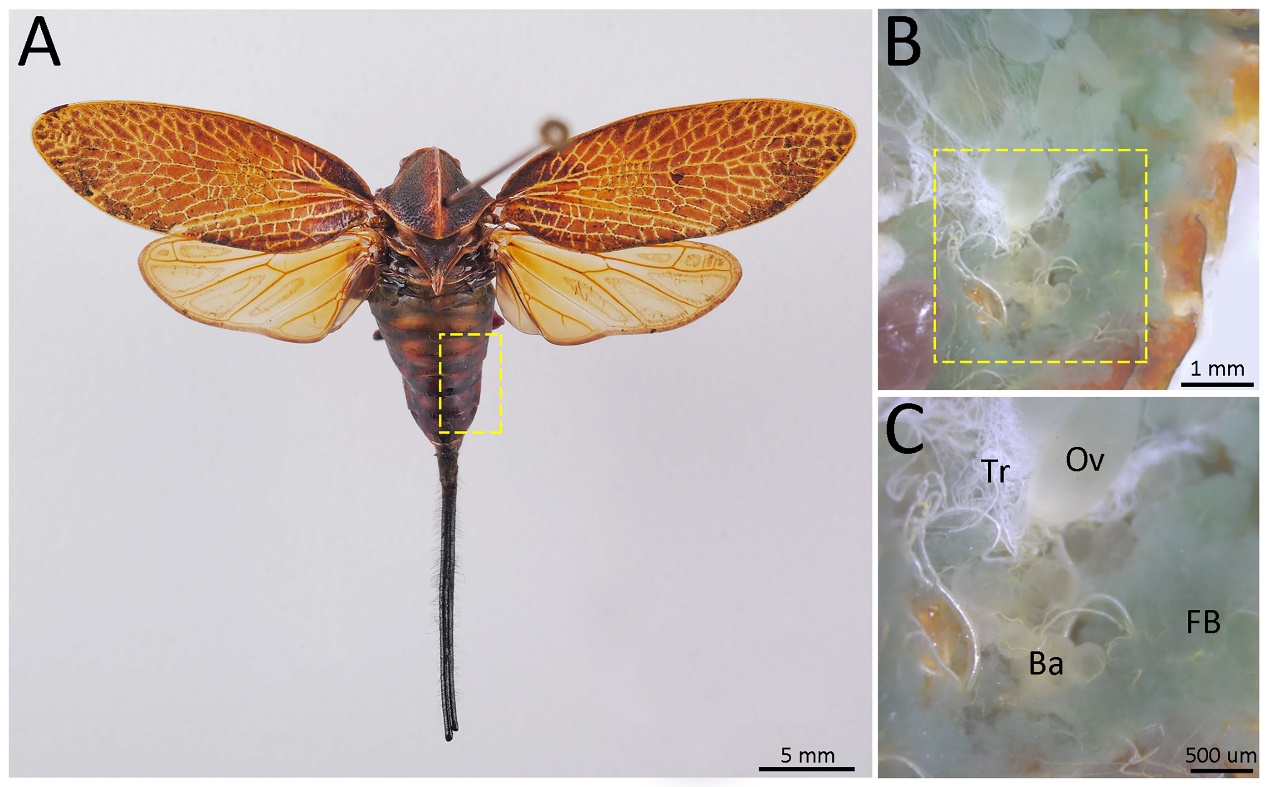


**Fig. S1** The position and morphology of bacteriomes, fat bodies and ovaries of treehopper *Darthula hardwickii*. (A) Habitus of female adult. (B) Anatomy of the right side of abdomen. (C) A magnified image corresponding to the yellow rectangle in (B). Ba, bacteriome; FB, fat body; Ov, ovary; Tr, tracheole.

**
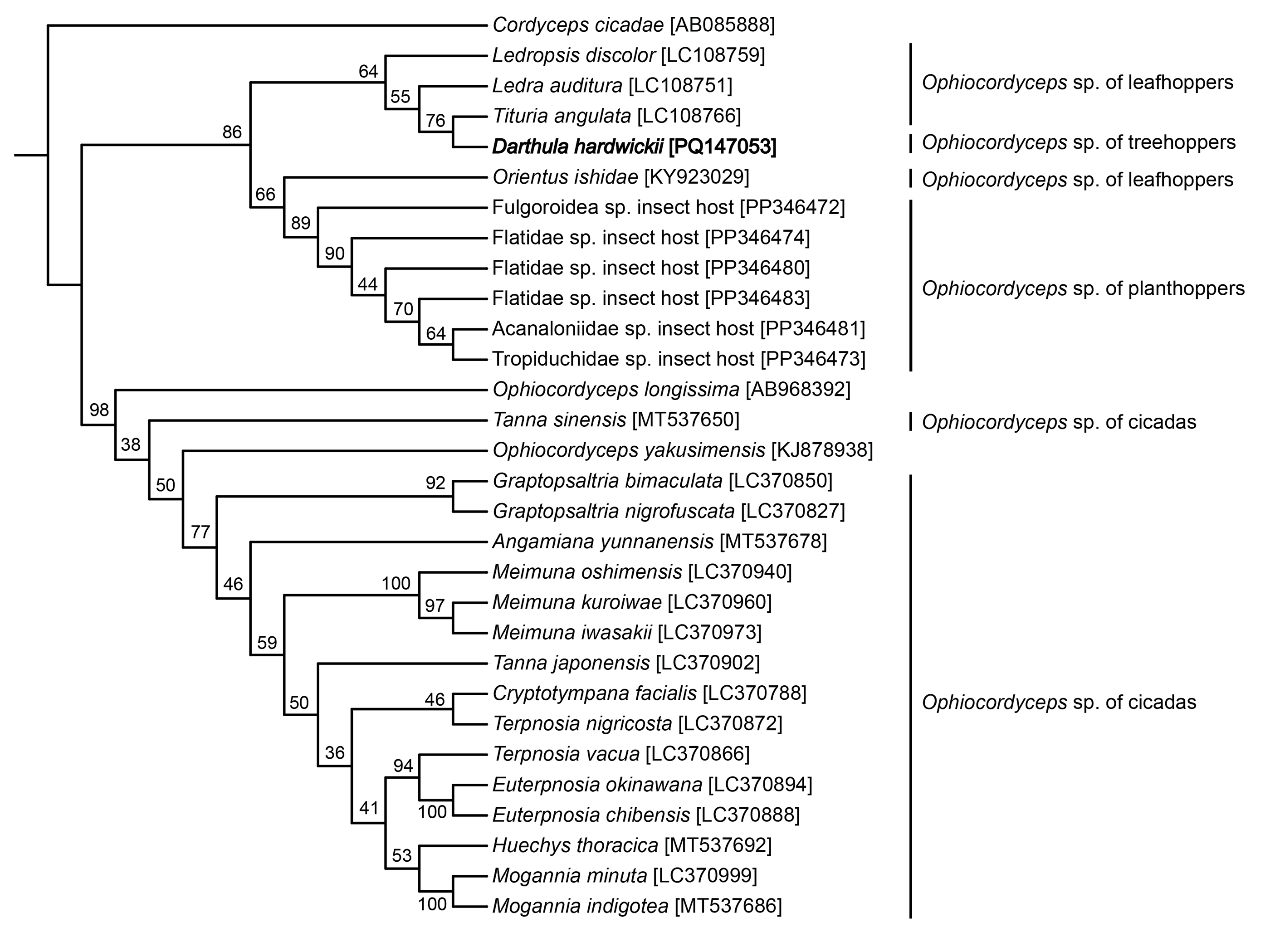
**

**Fig. S2** Phylogeny of YLS and its allies of *Ophiocordyceps* based on 18S rRNA sequence. The topology shows the best tree from Maximum-likelihood analysis. The sequence obtained in this study is indicated in bold black.


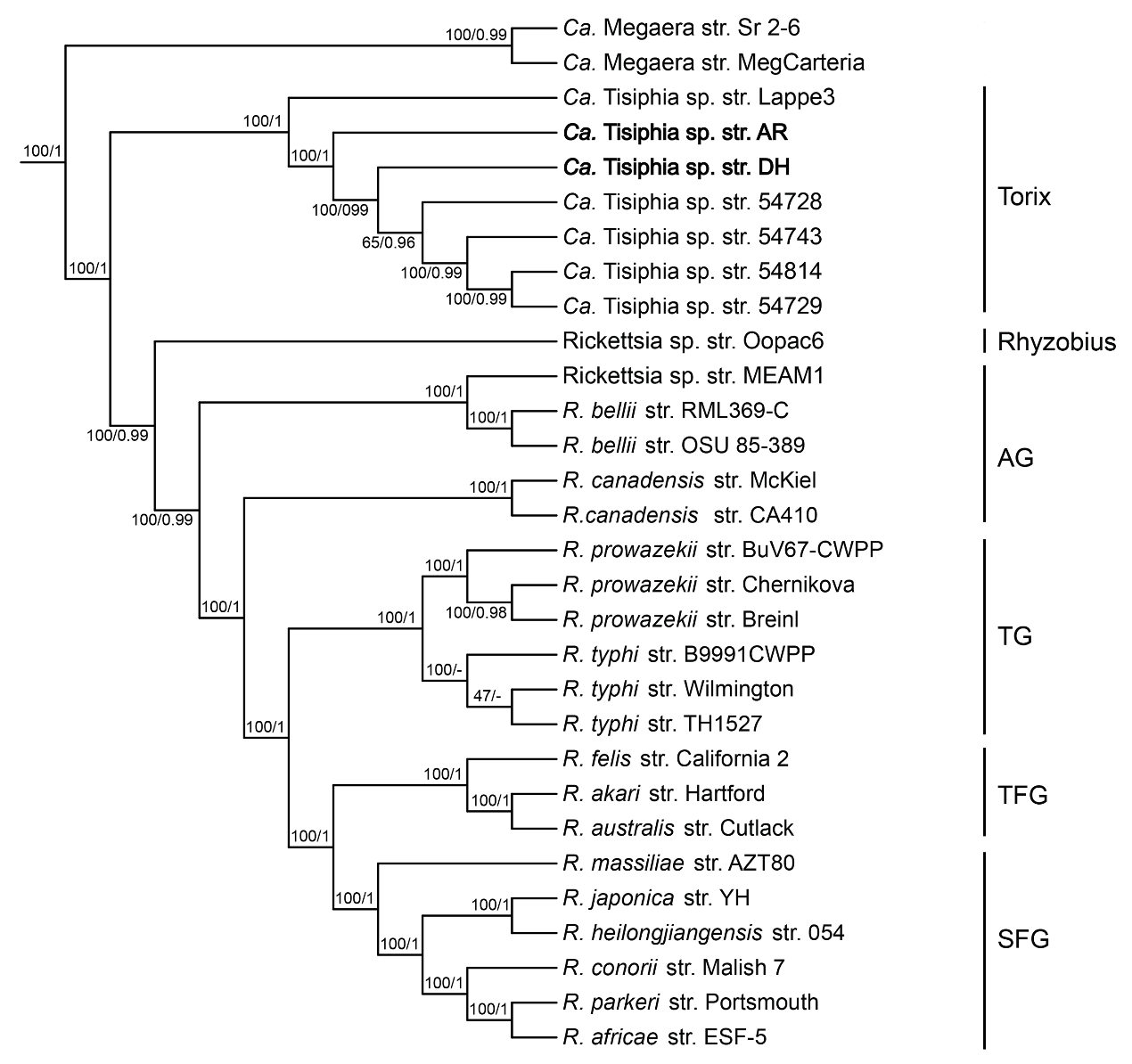


**Fig. S3** Maximum likelihood phylogeny of *Tisiphia* and its allies of Rickettsiales based on 16S rRNA gene and 364 single-copy orthologous genes. Bootstrap support values are indicated on each node in the order of maximum-likelihood/Bayesian inference. Terminals of the branch are labeled with available strain names. The sequence obtained in this study are indicated in bold black.


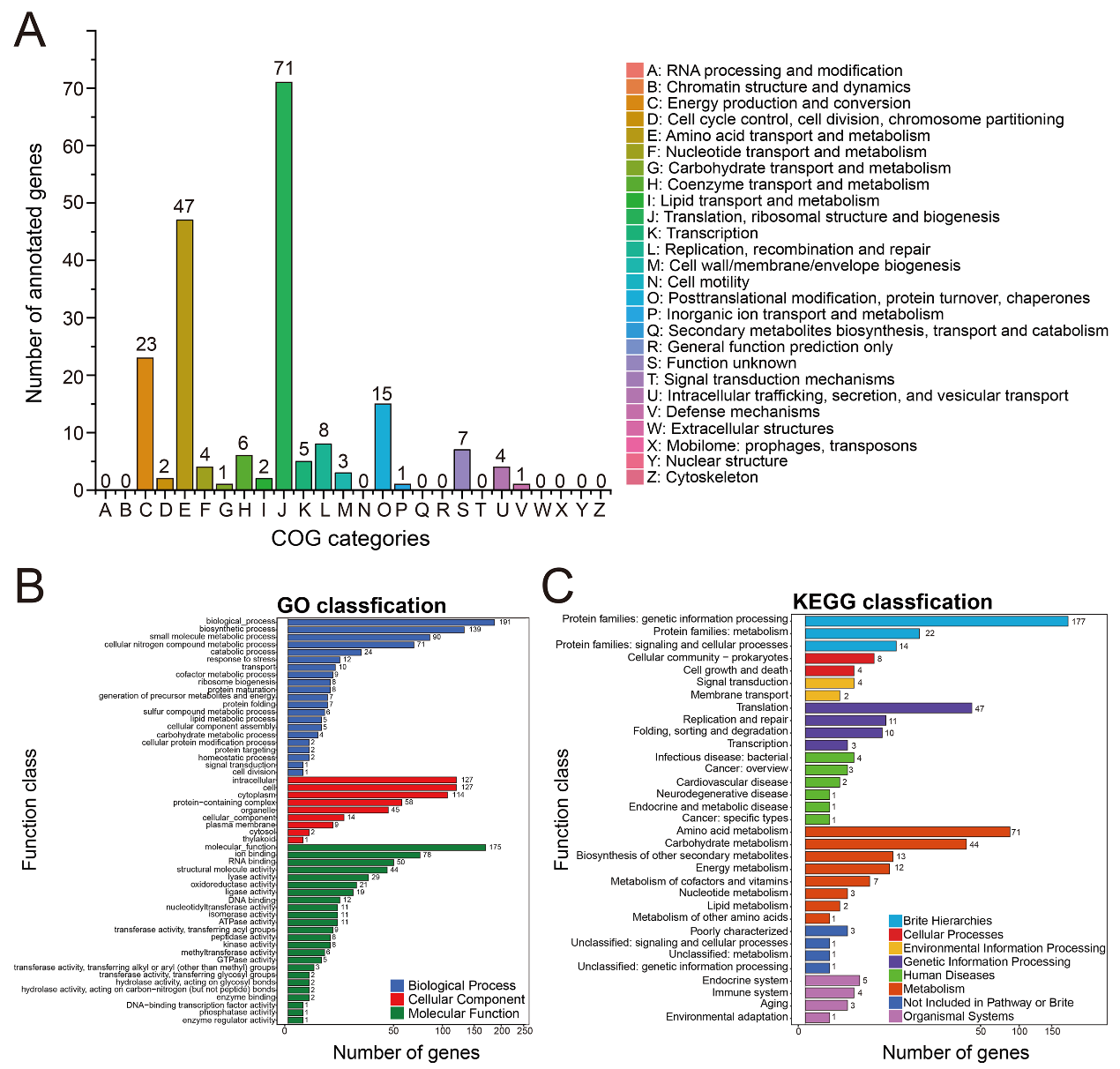


**Fig. S4** Functional category analysis of the *Karelsulcia*-DH annotated with the COG, GO, and KEGG databases. (A) COG annotation. The COG functional annotations are divided into 26 categories. (B) GO annotation. The GO functional classes are divided into three categories. (C) KEGG annotation. The KEGG functional classes are categorized into eight major categories.


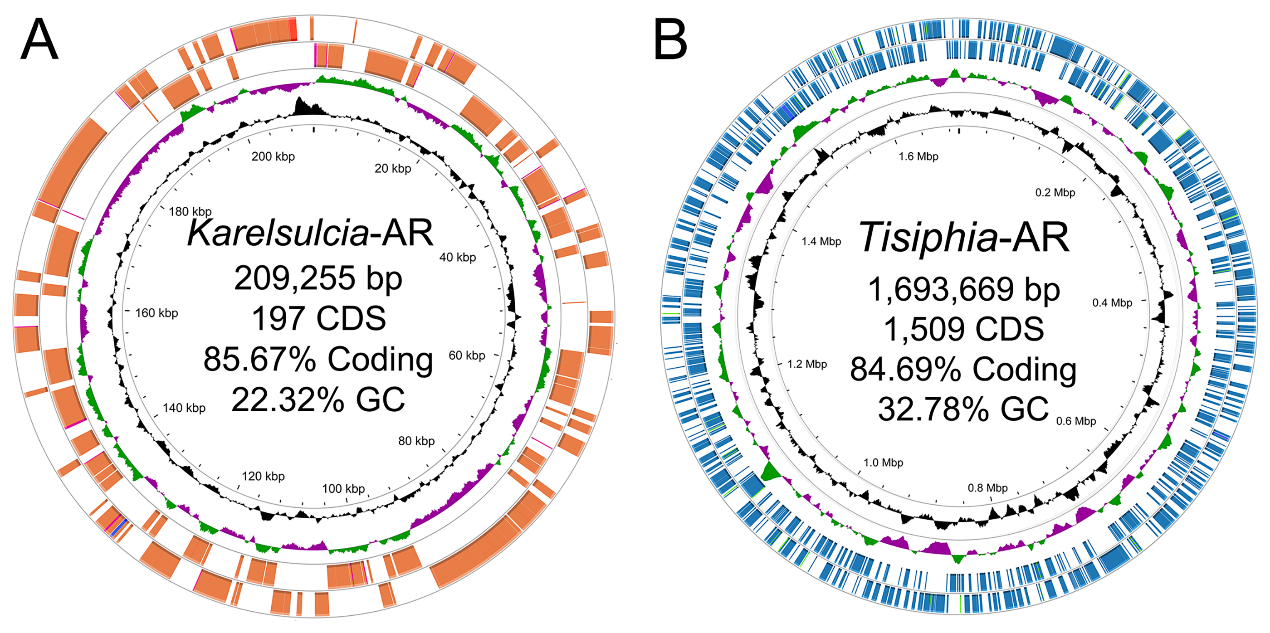


**Fig. S5** Circular genome maps of *Karelsulcia*-AR and *Tisiphia*-AR. (A, B) The four circles (outer to inner) represent the following. Circle 1 and 2 exhibit CDS on the forward and reverse strands, as well as rRNA, tRNA, and assembly gap; Circle 3 represents the GC skew ((C − G) / (C + G)) curve (dark green, positive GC skew; violet, negative GC skew); Circle 4 shows the GC content.


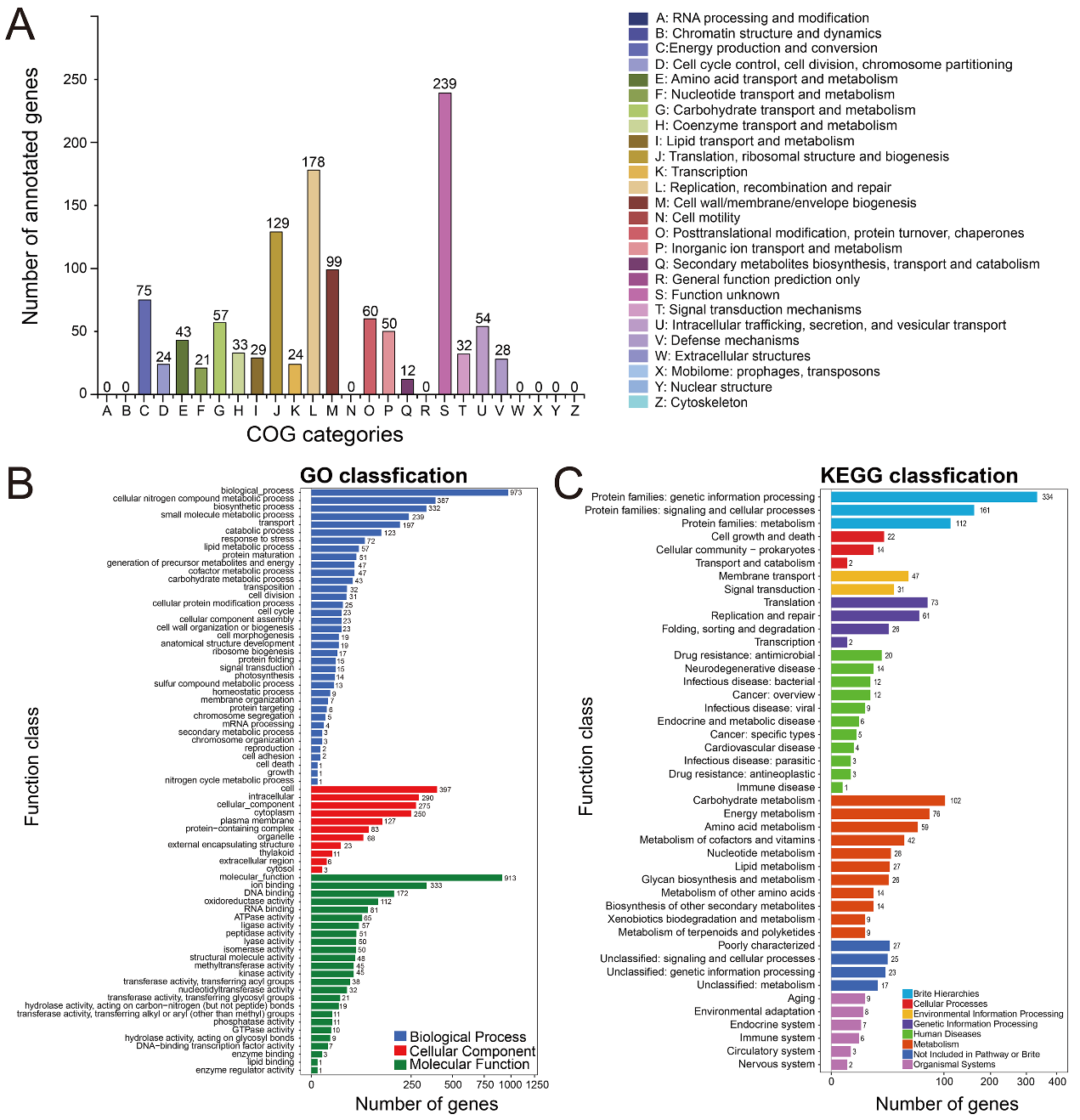


**Fig. S6** Functional category analysis of the *Tisiphia*-DH annotated with the COG, GO, and KEGG databases. (A) COG annotation. The COG functional annotations are divided into 26 categories. (B) GO annotation. The GO functional classes are divided into three categories. (C) KEGG annotation. The KEGG functional classes are categorized into eight major categories.


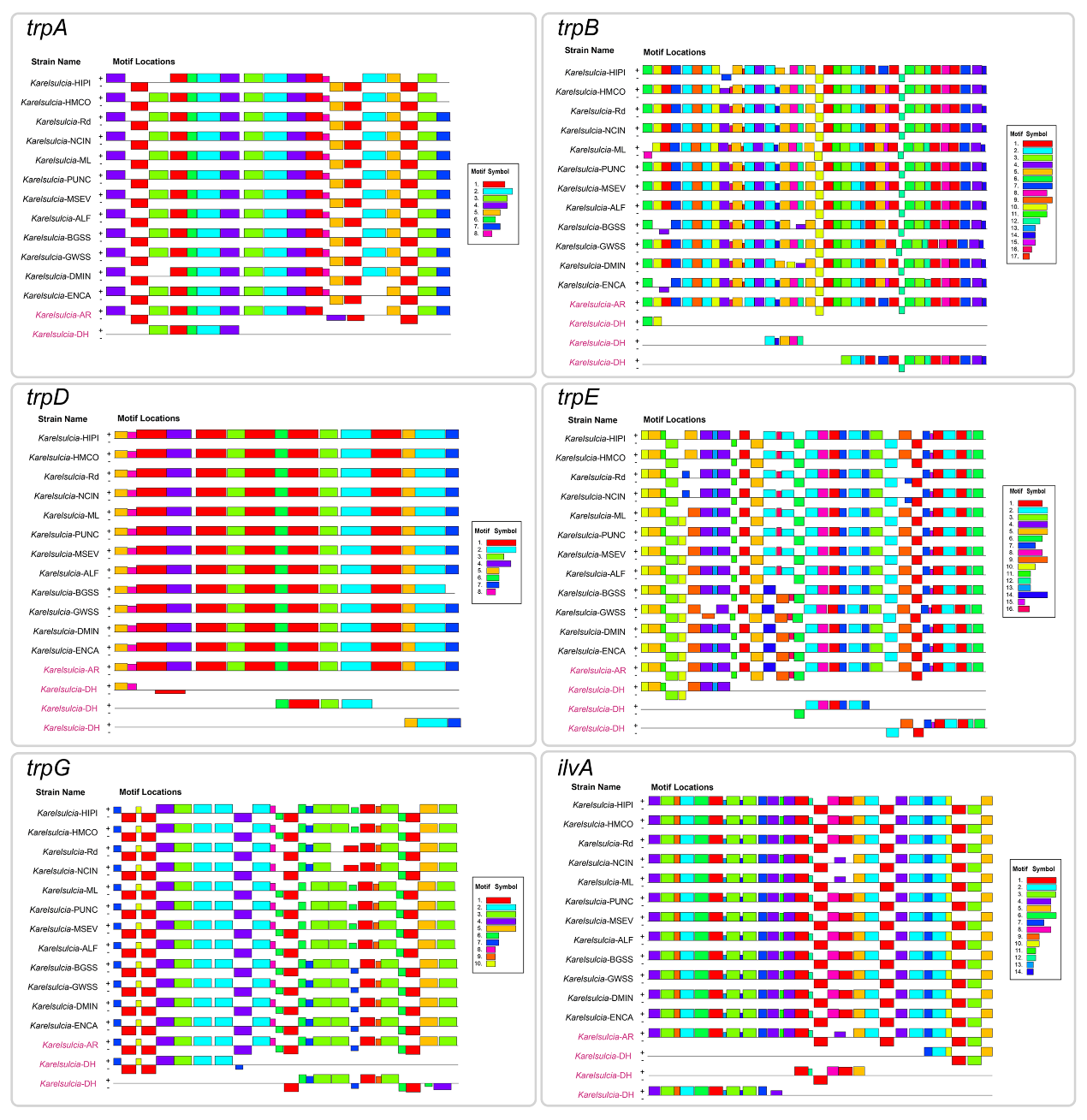


**Fig. S7** Comparison of truncated genes in *Karelsulcia*-DH and intact genes in 13 other sampled *Karelsulcia* lineages of treehoppers and leafhoppers. Label uses available strain names. The genes of *Karelsulcia*-DH and *Karelsulcia*-AR obtained in this study are indicated in pink. Motifs are represented by different colors and sizes. Up and down motifs are located on the positive and negative chains, respectively.

**Table S1** Probes used for fluorescence *in situ* hybridization.

| **Target** | **Sequence (5'-3')** | **Fluorochrome** | **References** |
| --- | --- | --- | --- |
| *Karelsulcia* | CCACACATTCCAGTTACTCC | CY3 | (1) |
| *Karelsulcia*-Lhelper | GTTCTGTGTGATCTCTATGCATTTCACCGCT | unlabelled |  |
| *Karelsulcia*-Rhelper | CCTCACTCTAGTTTATCAGTATCAATAGCACTT | unlabelled |  |
| YLS | CCTGCCTGGAGCACTCT | CY5 | (2) |
| YLS*-*Lhelper | CTAATGTATTCGAGCAT | unlabelled | (3) |
| YLS*-*Rhelper | TTTTTCAAAGTAAAAGTCCCGT | unlabelled |  |

1. Łukasik P, Nazario K, Van Leuven JT, Campbell MA, Meyer, M. 2018. Multiple origins of interdependent endosymbiotic complexes in a genus of cicadas. Proc Natl Acad Sci U S A 115:E226–E235.

2. Matsuura Y, Moriyama M, Łukasik P, Vanderpool D, Tanahashi M, Meng XY, McCutcheon JP, Fukatsu T. 2018. Recurrent symbiont recruitment from fungal parasites in cicadas. Proc Natl Acad Sci U S A 115:E5970–E5979.

3. Huang Z, Zhou J, Zhang Z, He H, Wei C. 2023. A study on symbiotic systems of cicadas provides new insights into distribution of microbial symbionts and improves fluorescence *in situ* hybridization technique. Int J Mol Sci 24:2434.

| **Target** | **Primer name** | **Product size** | **Sequence (5'-3')** | **Annealing temperature** | **References** |
| --- | --- | --- | --- | --- | --- |
| *16S rRNA* (*Karelsulcia*) | 10_CFB_FF | 1.5 kb | AGAGTTTGATCATGGCTCAGGATG | 58 °C | (1) |
|  | 1515_R |  | GTACGGCTACCTTGTTACGACTTAG |  |  |
| *16S rRNA* (*Tisiphia*) | Tis_F | 1.5 kb | GTAATCGCGGATCAGCATGC | 58 °C | this study |
|  | Tis_R |  | GATGAGCCCGCGTCAGATTA |  |  |
| *18S rRNA* (YLS) | Fng18S_82F | 1.0–1.5 kb | GAAACTGCGAATGGCT | 60-50 °C | (2) |
|  | Fng18S_1067R |  | TMTCGTAAGGTGCCGA | (for step) |  |

**Table S2** Primers used for PCR amplification.

1. Moran NA, Tran P, Gerardo NM. 2005. Symbiosis and insect diversification: an ancient symbiont of sap-feeding 11 insects from the bacterial phylum Bacteroidetes. Appl Environ Microbiol 71:8802–8810.

2. Matsuura Y, Moriyama M, Łukasik P, Vanderpool D, Tanahashi M, Meng XY, McCutcheon JP, Fukatsu T. 2018. Recurrent symbiont recruitment from fungal parasites in cicadas. Proc Natl Acad Sci U S A 115:E5970–E5979.

**Table S3** The information of public *Karelsulcia* species (retrieved from NCBI and reference).

| **Host species** | **Strain** | **Accession number or sources of reference** |
| --- | --- | --- |
| **Fulgoroidea** | | |
| *Oliarus filicicola* | OLIH | CP028359 |
| *Melanoliarus placitus* | / | (1) |
| **Cercopoidea** | | |
| *Philaenus spumarius* | PSPU | AP013293 |
| *Clastoptera arizonana* | CARI | CP002163 |
| **Cicadoidea** | | |
| *Auritibicen japonicus* | SMAURJAP | CP029021 |
| *Vagitanus terminalis* | SMVAGTER | CP029022 |
| *Meimuna oshimensis* | SMMEIOSH | CP029028 |
| *Euterpnosia chibensis* | SMEUTCHI | CP029011 |
| *Graptopsaltria nigrofuscata* | SMGRANIG | CP029013 |
| *Diceroprocta semicincta* | SMDSEM | CP001605 |
| *Hyalessa maculaticollis* | SMHYAMAC | CP029014 |
| *Muda kuroiwae* | SMMUDKUR | CP029017 |
| *Platypleura kaempferi* | SMPLAKAE | CP029019 |
| *Magicicada tredecim* | SMMAGTRE | CP010828 |
| *Kosemia yezoensis* | SMKOSYEZ | CP029015 |
| *Tettigades undata* | TETUND | CP007234 |
| *Okanagana oregona* | SMOKAORE | CP119458 |
| *Chilecicada* sp. | / | (1) |
| *Tettigades auropilosa* | / | (1) |
| *Okanagana villosa* | / | (1) |
| **Membracoidea** | | |
| *Homalodisca vitripennis* | GWSS | CP000770 |
| *Graphocephala atropunctata* | BGSS | CP008986 |
| *Draeculacephala minerva* | DMIN | CP001981 |
| *Macrosteles quadripunctulatus* | PUNC | CP013212 |
| *Macrosteles* sp. | MSEV | CP060020 |
| *Macrosteles quadrilineatus* | ALF | CP006060 |
| *Dalbulus maidis* | ML | CP010105 |
| *Nesophrosyne haleakala* | HMCO | CP093903 |
| *Nesophrosyne montium* | HIPI | CP093904 |
| *Nephotettix cincticeps* | NCIN | AP028057 |
| *Recilia dorsalis* | Rd | CP129521 |
| *Aphrodes bicincta* | / | (1) |
| *Graphocephala atropunctata* | / | (1) |
| *Graphocephala fennahi* | / | (1) |
| *Kolla paulula* | / | (1) |
| *Vidanoana flavomaculata* | / | (1) |
| *Cuerna arida* | / | (1) |

**Table S3** *continued*

| **Host species** | **Strain** | **Accession number or sources of reference** |
| --- | --- | --- |
| *Tinobregmus viridescens* | / | (1) |
| *Euscelidius variegatus* | / | (1) |
| *Agudus* sp. | / | (1) |
| *Penthimia* sp. | / | (1) |
| *Idiocerus rotundens* | / | (1) |
| *Macropsis decisa* | / | (1) |
| *Ponana quadralaba* | / | (1) |
| *Penestragania robusta* | / | (1) |
| *Hespenedra chilensis* | / | (1) |
| *Neocoelidia tumidifrons* | / | (1) |
| *Nionia palmeri* | / | (1) |
| *Stenocotis depressa* | / | (1) |
| *Ulopa reticulata* | / | (1) |
| *Entylia carinata* | ENCA | CP021172 |
| *Centrotus cornutus* | / | (1) |
| *Nessorhinus gibberulus* | / | (1) |
| *Procyrta* sp. | / | (1) |
| *Heteronotus* sp. | / | (1) |
| *Notocera* sp. | / | (1) |
| *Enchenopa_latipes* | / | (1) |
| *Membracis_tectigera* | / | (1) |
| *Holdgatiella chepuensis* | / | (1) |
| *Amastris* sp. | / | (1) |
| *Cyphonia_clavata* | / | (1) |
| *Chelyoidea* sp. | / | (1) |
| *Microcentrus caryae* | / | (1) |
| *Lycoderes burmeisteri* | / | (1) |
| *Lophyraspis* sp. | / | (1) |
| *Llanquihuea pilosa* | / | (1) |
| *Mapuchea* sp. | / | (1) |

1. Cao Y, Dietrich CH. 2021. Phylogenomics of flavobacterial insect nutritional endosymbionts with implications for Auchenorrhyncha phylogeny. Cladistics 38:38–58.

**Table S4** The information of public *Tisiphia* species and its allies of Rickettsiales (retrieved from NCBI).

| **Group** | **Strain** | **Size(bp)** | **GC content (%)** | **Accession number** |
| --- | --- | --- | --- | --- |
| *Ca.* Tisiphia | Lappe3 | 1,372,489 | 33 | JAJIYP000000000 |
| *Ca.* Tisiphia | 54728 | 1,360,024 | 32.5 | NZ_OZ034997 |
| *Ca.* Tisiphia | 54743 | 1,654,047 | 33 | NZ_OZ034919 |
| *Ca.* Tisiphia | 54729 | 1,458,157 | 33 | NZ_OZ032164 |
| *Ca.* Tisiphia | 54814 | 1,529,368 | 32.5 | NZ_OZ032144 |
| *Ca.* Megaera | MegCarteria | 1,298,707 | 34 | JAJIYJ000000000 |
| *Ca.* Megaera | Sr 2-6 | 1,862,567 | 35 | JARJFB000000000 |
| *Rickettsia* sp. | Oopac6 | 1,496,711 | 34.5 | JAJIYK000000000 |
| *Rickettsia* sp. | MEAM1 | 1,221,534 | 32 | AJWD00000000 |
| *Rickettsia* bellii | OSU 85-389 | 1,528,980 | 31.5 | NC_009883 |
| *Rickettsia* bellii | RML369-C | 1,522,076 | 31.5 | NC_007940 |
| *Rickettsia* canadensis | CA410 | 1,150,228 | 31 | NC_016929 |
| *Rickettsia* canadensis | McKiel | 1,159,772 | 31 | NC_009879 |
| *Rickettsia* prowazekii | BuV67-CWPP | 1,111,445 | 29 | NC_017056 |
| *Rickettsia* prowazekii | Breinl | 1,109,301 | 29 | NC_020993 |
| *Rickettsia* prowazekii | Chernikova | 1,109,804 | 29 | NC_017049 |
| *Rickettsia* typhi | TH1527 | 1,112,372 | 29 | NC_017066 |
| *Rickettsia* typhi | B9991CWPP | 1,112,957 | 29 | NC_017062 |
| *Rickettsia* typhi | Wilmington | 1,111,496 | 29 | NC_006142 |
| *Rickettsia* felis | California 2 | 1,485,148 | 32.5 | NC_007109 |
| *Rickettsia* australis | Cutlack | 1,323,280 | 32.5 | NC_017058 |
| *Rickettsia* akari | Hartford | 1,231,060 | 32.5 | NC_009881 |
| *Rickettsia* massiliae | AZT80 | 1,278,719 | 32.5 | NC_016931 |
| *Rickettsia* japonica | YH | 1,283,087 | 32.5 | NC_016050 |
| *Rickettsia* heilongjiangensis | 054 | 1,278,471 | 32.5 | NC_015866 |
| *Rickettsia* conorii | Malish 7 | 1,268,755 | 32.5 | NC_003103 |
| *Rickettsia* parkeri | Portsmouth | 1,300,386 | 32.5 | NC_017044 |
| *Rickettsia* africae | ESF-5 | 1,290,917 | 32.5 | NC_012633 |

**Table S5** The information of public YLSs and its allies of *Ophiocordyceps* (retrieved from NCBI).

| **Species** | **Host species** | **Host taxon** | **Accession number** |
| --- | --- | --- | --- |
| *Cordyceps cicadae* | Graptopsaltria nigrofuscata | cicadas | AB085888 |
| *Ophiocordyceps* sp. | *Tituria angulata* | leafhoppers | LC108766 |
| *Ophiocordyceps* sp. | *Ledropsis discolor* | leafhoppers | LC108759 |
| *Ophiocordyceps* sp. | *Ledra auditura* | leafhoppers | LC108751 |
| *Ophiocordyceps* sp. | *Orientus ishidae* | leafhoppers | KY923029 |
| *Ophiocordyceps* sp. | Fulgoroidea sp. insect host | planthoppers | PP346472 |
| *Ophiocordyceps* sp. | Flatidae sp. insect host | planthoppers | PP346474 |
| *Ophiocordyceps* sp. | Flatidae sp. insect host | planthoppers | PP346480 |
| *Ophiocordyceps* sp. | Flatidae sp. insect host | planthoppers | PP346483 |
| *Ophiocordyceps* sp. | Acanaloniidae sp. insect host | planthoppers | PP346481 |
| *Ophiocordyceps* sp. | Tropiduchidae sp. insect host | planthoppers | PP346473 |
| *Ophiocordyceps* sp. | *Tanna sinensis* | cicadas | MT537650 |
| *Ophiocordyceps* sp. | *Graptopsaltria bimaculata* | cicadas | LC370850 |
| *Ophiocordyceps* sp. | *Graptopsaltria nigrofuscata* | cicadas | LC370827 |
| *Ophiocordyceps* sp. | *Angamiana yunnanensis* | cicadas | MT537678 |
| *Ophiocordyceps* sp. | *Meimuna oshimensis* | cicadas | LC370940 |
| *Ophiocordyceps* sp. | *Meimuna kuroiwae* | cicadas | LC370960 |
| *Ophiocordyceps* sp. | *Meimuna iwasakii* | cicadas | LC370973 |
| *Ophiocordyceps* sp. | *Tanna japonensis* | cicadas | LC370902 |
| *Ophiocordyceps* sp. | *Cryptotympana facialis* | cicadas | LC370788 |
| *Ophiocordyceps* sp. | *Terpnosia nigricosta* | cicadas | LC370872 |
| *Ophiocordyceps* sp. | *Terpnosia vacua* | cicadas | LC370866 |
| *Ophiocordyceps* sp. | *Euterpnosia okinawana* | cicadas | LC370894 |
| *Ophiocordyceps* sp. | *Euterpnosia chibensis* | cicadas | LC370888 |
| *Ophiocordyceps* sp. | *Huechys thoracica* | cicadas | MT537692 |
| *Ophiocordyceps* sp. | *Mogannia minuta* | cicadas | LC370999 |
| *Ophiocordyceps* sp. | *Mogannia indigotea* | cicadas | MT537686 |
| *Ophiocordyceps longissima* | */* | / | AB968392 |
| *Ophiocordyceps yakusimensis* | */* | / | KJ878938 |
